# Detecting Key Functional Components Group and Speculating the Potential Mechanism of Xiao-Xu-Ming Decoction in Treating Stroke

**DOI:** 10.3389/fcell.2022.753425

**Published:** 2022-05-12

**Authors:** Yu-peng Chen, Ke-xin Wang, Jie-qi Cai, Yi Li, Hai-lang Yu, Qi Wu, Wei Meng, Handuo Wang, Chuan-hui Yin, Jie Wu, Mian-bo Huang, Rong Li, Dao-gang Guan

**Affiliations:** ^1^ Department of Biochemistry and Molecular Biology, School of Basic Medical Sciences, Southern Medical University, Guangzhou, China; ^2^ Guangdong Provincial Key Laboratory of Single Cell Technology and Application, Southern Medical University, Guangzhou, China; ^3^ Guangdong Provincial Key Laboratory on Brain Function Repair and Regeneration, Department of Neurosurgery, National Key Clinical Specialty/Engineering Technology Research Center of Education Ministry of China, Neurosurgery Institute, Zhujiang Hospital, Southern Medical University, Guangzhou, China; ^4^ Department of Radiology, Nanfang Hospital, Southern Medical University, Guangzhou, China; ^5^ Department of Burns, Nanfang Hospital, Southern Medical University, Guangzhou, China; ^6^ Department of Histology and Embryology, Guangdong Provincial Key Laboratory of Construction and Detection in Tissue Engineering, School of Basic Medical Sciences, Southern Medical University, Guangzhou, China; ^7^ Department of Cardiovascular Disease, First Affiliated Hospital of Guangzhou University of Chinese Medicine, Guangzhou, China

**Keywords:** XXMD, KFCG, network pharmacology, functional response space, effective proteins, PC12 cells, HT22 cells

## Abstract

Stroke is a cerebrovascular event with cerebral blood flow interruption which is caused by occlusion or bursting of cerebral vessels. At present, the main methods in treating stroke are surgical treatment, statins, and recombinant tissue-type plasminogen activator (rt-PA). Relatively, traditional Chinese medicine (TCM) has widely been used at clinical level in China and some countries in Asia. Xiao-Xu-Ming decoction (XXMD) is a classical and widely used prescription in treating stroke in China. However, the material basis of effect and the action principle of XXMD are still not clear. To solve this issue, we designed a new system pharmacology strategy that combined targets of XXMD and the pathogenetic genes of stroke to construct a functional response space (FRS). The effective proteins from this space were determined by using a novel node importance calculation method, and then the key functional components group (KFCG) that could mediate the effective proteins was selected based on the dynamic programming strategy. The results showed that enriched pathways of effective proteins selected from FRS could cover 99.10% of enriched pathways of reference targets, which were defined by overlapping of component targets and pathogenetic genes. Targets of optimized KFCG with 56 components can be enriched into 166 pathways that covered 80.43% of 138 pathways of 1,012 pathogenetic genes. A component potential effect score (PES) calculation model was constructed to calculate the comprehensive effective score of components in the components-targets-pathways (C-T-P) network of KFCGs, and showed that ferulic acid, zingerone, and vanillic acid had the highest PESs. Prediction and docking simulations show that these components can affect stroke synergistically through genes such as MEK, NFκB, and PI3K in PI3K-Akt, cAMP, and MAPK cascade signals. Finally, ferulic acid, zingerone, and vanillic acid were tested to be protective for PC12 cells and HT22 cells in increasing cell viabilities after oxygen and glucose deprivation (OGD). Our proposed strategy could improve the accuracy on decoding KFCGs of XXMD and provide a methodologic reference for the optimization, mechanism analysis, and secondary development of the formula in TCM.

## Introduction

In the treatment of complex diseases, TCM usually work in the form of multicomponents and multi-targets, and these components and targets could form complex network. This network contains effective action, side effective and toxicity networks. How to extract the effective action network and obtain the KFCG is the key goal of the optimization of prescriptions in TCM.

At present, methods have been established by decoding the potential mechanisms of prescription in treating complex disease, including collections of Chinese medicine components, active ingredients screening, targets prediction, pathways analyzations, and inference of the mechanism of targets. These analysis methods have successfully parsed the mechanisms of some treatments of prescriptions in TCM on the complex disease. For example, Pan et al. used the network pharmacology model and experimental validation to confirm that quercetin in Huanglian Decoction (HLD) affected the synthesis of glucose transporter 4 (GLUT4) by interfering with the insulin signaling pathway and improved the therapeutic effect of HLD on type 2 diabetes (T2DM) (Pan et al., 2020). Xu et al. found that Yinlai Decoction (Y D) regulated the expression of inflammatory factor IL-6, mediated the host’s immune inflammatory response, and reduced the symptoms and signs of pneumonia by using the network pharmacology model (Xu J. et al., 2020). Zhang et al. found that Ermiao fang (EMF) inhibited the activities of proteins in the NF-κB and MAPK pathways of rats with endometritis, and EMF played an anti-inflammatory role in the treatment of endometritis (Zhang et al., 2020b).

However, most of current analyses are based on drug-targets or pathogenetic genes. There is a lack of the quantification of network propagation of the intervention effects between drug-targets and pathogenetic genes. Thus, it is desirable to design a novel strategy to solve these problems.

Stroke is a cerebrovascular event with cerebral blood flow interruption which is caused by occlusion or bursting of cerebral vessels. It can cause multiple functional damage of body and sustain with some complications (An et al., 2019; Liu et al., 2021). In stroke and poststroke, massive formation of inflammatory cytokines glycoproteins and monocyte chemoattractant proteins (McKimmie and Graham, 2010) may cause neuronal death in different brain regions (Miyawaki et al., 2008; Ofengeim et al., 2012), In particular, neuron damage in hippocampus (hippocampal stroke) may lead to cognitive dysfunction (Liu et al., 2005; Biessels and Reagan, 2015). Besides emergency clinical surgery, treatment of stroke aims to relieve poststroke disorders, including the methods of the recovery of cerebrovascular injury, cognitive deficits, and brain parenchymal cells (Tanaka et al., 2002; Hermann et al., 2015). Deeply, some of the compounds, such as edaravone, citicoline, fluoxetine, and niacin, are already at clinical use or being trialed at clinical issues (Szelenberger et al., 2020; Chen et al., 2021). In addition, some prescriptions of TCM were being classically and widely used in China and some countries in Asia, such as XXMD (Wu et al., 2002; Wang and Xiong, 2012; Wang Y.-H. et al., 2019), Di-Tan Decoction (DTD) (Kwon et al., 2021), and Buyang Huanwu Tang (BYHWT) (Jin et al., 2019; Zhang Y. et al., 2020; Kook et al., 2021). Among them, XXMD is one of the prescriptions as an oral liquid in clinic applications throughout ancient and modern China (Cai et al., 2007; Fu et al., 2013; Luo X. et al., 2019; Jia et al., 2019). It can be applied on both ischemic stroke and hemorrhagic stroke as an important adjuvant treatment (Ye et al., 1999; An et al., 2019; Zhang et al., 2021). It is widely reported to be used in acute cerebral infarction (Cheng et al., 2019), acute atherosclerotic stroke (Zhou et al., 2021), and apoplectic hemiplegia (Hu, 2010). XXMD is also widely used in the combination therapy, including the combinations with alteplase (Liu and Qin, 2018), acupuncture (Ge, 2018), and herbal ingredients (Li and Cui, 2012; Wei and Wang, 2019). For example, it can significantly improve the infusion of the infarct center and its surrounding area in patients with acute cerebral infarction, especially in the infusion of infracted tissue with the combination with alteplase (Liu and Qin, 2018), significantly decrease the National Institute of Health stroke scale (NIHSS) of ischemic stroke patients in the combination with dipyridamole (Chang, 2015), and significantly increase the Fugl-Meyer motor functions and Barthel index in the stroke sequela phase of ischemic stroke patients in the combination with aspirin and nimodipine (Rui and Huang, 2018).

In addition, during the animal experiments, XXMD has been proved to keep mitochondrial function (Lan et al., 2018), inhibit neuroinflammation (Liu et al., 2021), and regulate lipid metabolism regulation (Jia et al., 2019), suggesting that XXMD plays an important role in the treatment of stroke. Modern pharmacological studies showed that XXMD improved the expression of MT-ND1 protein through protein hydrolysis and phosphatidylinositol signaling pathway, and protected brain mitochondrial homeostasis during chronic cerebral hypoperfusion in rats (Wang Y.-H. et al., 2019). However, there is still lack of the mechanism study of XXMD at the system level due to the characteristic of multicomponents and multi-targets in the prescriptions.

XXMD comprises 12 herbs: Ephedra alata Decne. (Mahuang, MH) (50 g), Stephania tetrandra S.Moore (Fangji, FJ) (50 g), Ginseng quinquefolium (L.) Alph.Wood (Renshen, RS) (50 g), Scutellaria baicalensis Georgi (Huangqin, HQ) (50 g), Cinnamomum cassia (L.) J.Presl (Rougui, RG) (50 g), Glycyrrhiza uralensis Fisch. (Gancao, GC) (50 g), Paeonia lactiflora Pall. (Baishao, BS) (50 g), Ligusticum striatum DC. (Chuanxiong, CX) (50 g), Amygdalus communis L. (Kuxingren, KXR) (50 g), Aconitum wilsonii Stapf ex Veitch (Fuzi, FZ), Saposhnikovia divaricata (Turcz.) Schischk. (Fangfeng, FF) (75 g), and Zingiber officinale Roscoe (Shengjiang, SJ) (250 g). In this prescription, MH (Huang et al., 2020) and FJ (Kong et al., 2019) have the function of anti-inflammatory; CX has antithrombotic and antiatherosclerotic activities (Ye et al., 1999); RS has a function of potential neuroprotection by promoting biological activities such as neurogenesis, anti-apoptosis, oxidative stress, energy supplementation, and cerebral circulation, and alleviating brain edema (Zhang et al., 2020a; Shi et al., 2020); HQ contains a large amount of flavones, such as baicalein, wogonin, and oroxylin A, and has analgesic, antipyretic, and antioxidant effects (Li C. et al., 2011). It is found in modern pharmacological studies that ginsenoside GRb1 in ginseng can affect the cAMP/PKA/CREB pathway and promote nerve axon regeneration (Liu B. et al., 2020); glycyrrhizic acid in GC can affect the TLR4/NF-κB pathway and reduce inflammatory damage (Yan et al., 2019); curcumin regulated the extracellular signal-regulated kinase ERK-mTOR signaling pathway and inhibited neural stem cell phagocytosis (Wang M. et al., 2019). These active components have a neuroprotective and anti-inflammatory effect on stroke treatment. However, the effective components of XXMD for stroke have only been sporadically reported (Liu et al., 2002; Wu et al., 2002; Wang and Xiong, 2012), and the prescription optimization of XXMD in treating stroke has been rarely reported (Lu et al., 2018).

In this study, we used the treatment of stroke with XXMD as an example to build a quantitative network pharmacology model. We studied the KFCG optimization and potential mechanism speculation of XXMD in the treatment of stroke systematically through the screening of TCM ingredients, component targeting analysis, model prediction, Gene Ontology (GO) and pathway enrichment analysis, docking simulations, and experimental validations. It can be superior in finding the KFCG of XXMD more comprehensively and can explain the relationship between stroke pathogenetic genes and targets more clearly. Our strategy could provide a methodologic reference for prescription optimization and secondary development.

## Materials and Methods

### Pathogenetic Genes

We query stroke in DisGeNET and obtain a total of 14 stroke-related IDs that are consistent with the IDs of international classification of diseases (ICD10). The IDs are as follows: C0948008, C1857287, C3178801, C0242129, C0262469, C1112433, C1299567, C1298680, C0265113, C0038454, C0740392, C0751956, C0553692, and C3536593. Among these IDs, C0553692 is related to hemorrhagic stroke. The genes obtained from these above 14 IDs were treated as pathogenetic genes of stroke (Supplementary Table S1).

### Constructing Weighted Gene Regulatory Network Based on Pathogenetic Genes

To construct comprehensive weighted gene network of stroke, the protein–protein interactions (PPI) data were collected from public web servers CMGRN and PTHGRN (Guan et al., 2014a; Guan et al., 2014b). Pathogenetic genes with evidence number from DisGeNET (Pinero et al., 2017) were mapped to the PPI network and used in constructing the weighted gene regulatory network of stroke. Cytoscape (Version 3.7.2) was utilized to visualize the network.

### Components Collection of Xiao-Xu-Ming Decoction

All herbal components of XXMD were extracted from literatures and two published natural product databases: traditional Chinese medicine systems pharmacology database and analysis platform (TCMSP) (Ru et al., 2014) and the Encyclopedia of Traditional Chinese Medicine (ETCM) (Xu H.-Y. et al., 2019).

### Select Potential Active Components of Xiao-Xu-Ming Decoction

The potential active components of XXMD were selected from the TCMSP database and those detected in herbs. The properties of all components of XXMD were retrieved from TCMSP, including molecular weight (MW), oral bioavailability (O B) (Sugumaran et al.), Caco-2 permeability (Caco-2), and drug-likeness (Olson et al.) (Xu et al., 2012). Three absorption, distribution, metabolism, and excretion-related (ADME-related) models, including OB (Sugumaran et al.), Caco-2, and DL, were employed to screen the components. Components with the properties of OB ≥ 30%, Caco-2> −0.4, and DL ≥ 0.18 (Zhang et al., 2020c; Feng et al., 2021; Wang et al., 2021) were considered the potential active components. Owing to high concentration and high biological activities reported in literatures, some components in XXMD that did not meet ADME-screening criteria were also manually selected and used in conjunction with components from ADME screening in the following study (Table 1).

**TABLE 1:** The information on chemical analysis of the herbs from the literature in 12 herbs.

Herb	Method	Component	Concentration (mg/g)	Reference
BS	HPLC	(+)-catechin	0.03	Yang et al. (2019)
		Albiflorin	9.29	Deng et al. (2019)
		Benzoic acid	0.69	Wu et al. (2015)
		Benzoyl paeoniflorin	0.10	Wang et al. (2011)
		Benzoyloxypaeoniflorin	0.20	—
		Benzoylpaeoniflorin	2.58	—
		Gallic acid	6.41	—
		Galloylpaeoniflorin	0.29	—
		Mudanpioside F	0.26	—
		Oxypaeonidanin	0.62	—
		Oxypaeoniflorin	12.41	—
		Paeoniflorin	27.62	—
		Paeonol	0.07	—
		Pentagalloylglucose	4.80	—
CX	UHPLC–MS/MS, HPLC, UHPLC–MS/MS	3,5-O-dicaffeoylquinic acid	0.62	Liang et al. (2019)
	3-butyl-1(3H)-Isobenzofuranone	0.23	Wang et al. (2020c)
	3-Butylidenephthalide	0.98	—
	Butylidenephthalide	0.77	—
	Butylphthalide	0.15	—
	Caffeic acid	0.02	—
	Chlorogenic acid	0.33	—
	Coniferyl ferulate	2.69	—
	Cryptochlorogenic acid	0.60	—
	Ferulic acid	0.19	—
	Gallic acid	0.03	—
	Levistolide-A	0.95	—
	L-tryptophan	0.03	—
	Neocnidilide	0.49	—
	Protocatechuic acid	0.05	—
	Senkyunolide A	9.59	—
	Senkyunolide H	0.13	—
	Senkyunolide I	0.74	—
	Tetramethylpyrazine	0.17	—
	Vanillic acid	0.08	—
	Vanillin	0.51	—
	Z-ligustilide	14.17	—
FF	HPLC	4′-O-glucosyl-5-O-methylvisamminol	4.40	Li et al. (2010)
	5-O-methylvisammioside	3.22	Li et al. (2011b)
	Ammijin	0.11	Zhao et al. (2013)
	Cimifugin	1.16	—
	Prim-O-glucosylcimifugin	1.48	—
	Sec-O-glucosylhamaudol	0.32	—
FJ	RP-HPLC	Fangchinoline	14.30	Lu et al. (2015)
	Tetrandrine	18.00		
FZ	UHPLC, HPLC	Aconitine	0.17	He et al. (2017)
		Benzoylaconine	0.39	Tang et al. (2013)
		Benzoylhypaconine	0.38	—
		Benzoylmesaconine	1.80	—
		Crassicauline A	0.15	—
		Dopamine hydrochloride	0.15	—
		Guanosine	0.24	—
		Hypaconitine	0.33	—
		Mesaconitine	0.49	—
		Salsolinol	1.33	—
		Uracil	0.01	—
		Uridine	0.38	—
		Yunaconitine	0.84	—
GC	HPLC	Echinatin	0.80	Wang et al. (2020a)
		Formononetin	0.39	Zhang et al. (2013)
		Glycyrrhetic acid	15.32	Hou et al. (2018)
		Glycyrrhizin	18.23	Zhang et al. (2019b)
		Isoliquiritigenin	2.23	Fang et al. (2016)
		Isoliquiritin	1.10	Shu et al. (2013)
		Isoliquiritin apioside	13.00	Chen et al. (2017a)
		Licochalcone B	2.00	—
		Licorice-saponin G2	4.50	—
		Liquiritigenin	0.75	—
		Liquiritin	8.23	—
		Liquiritin apioside	38.67	—
		Uralsaponin B	17.95	—
HQ	UHPLC, HPLC	1,2,3,4,6-pentagalloylglucose	3.41	Cui et al. (2016)
		Albiflorin	7.03	Guo et al. (2018)
		Apigenin	4.58	Zhuang et al. (2012)
		Apigenin-7-glucuronide	2.11	—
		Baicalein	20.20	—
		Baicalin	161.27	—
		Chrysin, aspen	2.26	—
		Gallic acid	3.11	—
		Oroxylin A	2.57	—
		Oroxylin A-7-O-glucuronide	11.30	—
		Paeoniflorin	16.10	—
		Scutellarein	1.61	—
		Scutellarin	2.78	—
		Wogonin	8.69	—
		Wogonoside	43.73	—
KXR	HPLC	Amygdalin	17.73	Shu et al. (2013)
		Benzoic acid	1.36	
MH	HPLC	Ephedrine	18.10	An et al. (2015)
		Ephedrine hydrochloride	12.17	Zhang et al. (2019d)
		Methylephedrine	3.00	—
		Methylephedrine hydrochloride	0.97	—
		Norephedrine	1.10	—
		Norpseudoephedrine	3.40	—
		Pseudoephedrine	8.90	—
		Pseudoephedrine hydrochloride	4.79	—
RG	RP-HPLC	Cinnamaldehyde	48.29	He et al. (2005)
	Cinnamic acid	0.47		
	Cinnamyl alcohol	1.77		
	Coumarin	0.85		
RS	LC-MS/MS, HPLC	Berberine	2.50	Filipiak-Szok et al. (2017)
		Brucine	0.50	Stavrianidi et al. (2017)
		Caffeic acid	10.30	Xu et al. (2011)
		Caffeine	14.20	Chen et al. (2016)
		Chlorogenic acid	5.80	Zhu et al. (2014)
		Ferulic acid	32.70	—
		Gallic acid	22.20	—
		Ginsenoside F2	0.17	—
		Ginsenoside R1	0.30	—
		Ginsenoside Rb1	3.99	—
		Ginsenoside Rb2	3.19	—
		Ginsenoside Rb3	1.76	—
		Ginsenoside Rc	5.31	—
		Ginsenoside Rd	8.84	—
		Ginsenoside Re	18.71	—
		Ginsenoside Rf	1.05	—
		Ginsenoside Rg1	9.46	—
		Ginsenoside Rg2	0.65	—
		Ginsenoside Rg3	0.21	—
		Ginsenoside Rh1	0.08	—
		Ginsenoside Rh2	0.71	—
		Ginsenoside Ro	4.84	—
		Harmine	2.90	—
		Hyperoside	3.10	—
		Kaempferol	1.10	—
		p-coumaric acid	10.50	—
		Quercetin	19.40	—
		Quercitrin	130.70	—
		Rhamnetin	1.90	—
		Rutin	22.00	—
		Theobromine	3.20	—
		Yohimbine	0.70	—
SJ	LC-MS	6-gingerol	106.80	Li et al. (2019b)
	6-shogaol	42.90		
	8-gingerol	53.20		
	8-shogaol	27.40		
	10-gingerol	37.40		
	10-shogaol	23.50		
	Zingerone	27.30		

### Predict Targets of Potential Active Components

To obtain the targets of potential active components in XXMD, Open Babel toolkit (version 2.41) was used in the conversion of all chemical structures into canonical SMILES. After that, the commonly used tools, i.e., Similarity Ensemble Approach (SEA) (Tao et al., 2013), HitPick (Liu et al., 2013), and SwissTarget Prediction (Gfeller et al., 2014), were employed to predict targets of potential active components based on canonical SMILES.

### Construction of Quantitative Network Pharmacology Model

Node importance is an important topological property and can be used to evaluate the influence of nodes among the network. The nodes whose node importance is larger than the average node importance of all nodes are treated as critical roles and hub nodes in the network (Liu et al., 2016). Here, we designed a novel node importance calculation method to figure out the importance and the influence of genes. According to this rule, these nodes with higher importance scores than average importance score are kept and integrated with their edges to form functional response space (FRS). The detail of the method is described as follows:

$${\mathrm{Nim}}_{\mathrm{(s)}}\;=\;\sqrt[{2}]{\left[\sum_{s\;\neq v\;\neq t\;\in V}\frac{\sigma_{vt\;\left(s\right)}}{\sigma_{vt}}\;\right]\;\times \sum_{s\neq x}\frac{1}{d_{\left(s,\;x\right)}}}$$

Nim represents the node importance; $\sigma_{vt}$ represents the number of the shortest path between node *v* and node *t*, $\sigma_{vt\;\left(s\right)}$ is the number of the shortest path passing through node s from node *v* to *t*; *x*, *v* represent nodes (genes); $d_{\left(s,x\right)}$ represents the shortest distance (minimum number of edges) when *s* and *x* are connected.

After being quantized, Nim(s)  was sorted from small to large, and was represented by a new variable Q.

$$Q=\left[Q_{1},\;Q_{2},\;Q_{3},\;\ldots ,\;Q_{\left|\;V\;\right|}\right]=\;\left[\mathrm{Nim}{\left(s\right)}_{x},\;\mathrm{Nim}{\left(s\right)}_{x+1},\;\mathrm{Nim}{\left(s\right)}_{x+2},\;\ldots ,\;\mathrm{Nim}{\left(s\right)}_{x+n}\right],\;x\in \left[1,\;\left|\;V\;\right|\right]\;\mathrm{and}\;x+n=\left|\;V\;\right|$$

The new variable G represented the nodes in the network. Each G responds to its unique Q.

$$G\;\in \;\left\{G_{1},\;G_{2},\;G_{3},\;\ldots ,\;G_{\left|\;V\;\right|}\right\}\;⇌\;\left[Q_{1},\;Q_{2},\;Q_{3},\;\ldots ,\;Q_{\left|\;V\;\right|}\right]$$

FRS represent the set of genes that were selected with our proposed method from the unite of pathogenetic genes and XXMD targets. N represented natural number. *g* represented a node *g*.

$$\mathrm{FRS}\;\in \{\begin{array}{c} \left\{G_{g}\;,\;\ldots ,G_{\left(\left|\;V\;\right|-2\right)}\;,\;G_{\left(\left|\;V\;\right|-1\right)}\;,\;G_{\left|\;V\;\right|}\right\}\;\\ ⇌\;\left[Q_{\left(\left|\;V\;\right|+1\right)/2}\;,\;\ldots ,Q_{\left(\left|\;V\;\right|-2\right)}\;,\;Q_{\left(\left|\;V\;\right|-1\right)}\;,\;Q_{\left|\;V\;\right|}\right],\;\;\;\;\left|\;V\;\right|=2p+1\;and\;p\in N\\ \left\{G_{g}\;,\ldots ,\;G_{\left(\left|\;V\;\right|-2\right)}\;,\;G_{\left(\left|\;V\;\right|-1\right)}\;,\;G_{\left|\;V\;\right|}\right\}\;\\ ⇌\;\left[\frac{\left(Q_{\left|\;V\;\right|/2}+\;Q_{\left(\left|\;V\;\right|+2\right)/2}\right)}{2}\;,\ldots ,\;Q_{\left(\left|\;V\;\right|-2\right)}\;,\;Q_{\left(\left|\;V\;\right|-1\right)}\;,\;Q_{\left|\;V\;\right|}\right],\;\;\;\;\left|\;V\;\right|=2p\;\;and\;p\in N \end{array}$$

### The Selection of Key Functional Components Group With Components Contribution Ratio Model

To optimize effective components and get the KFCG, which would be used to illustrate the potential molecular mechanism of XXMD in the therapy of stroke, we designed a components contribution ratio (CCR) model to select KFCG:

For x∈(1:n) do

if $W_{kx}=\max\;\left\{W_{k1}\;:\;W_{kn}\right\}$ then

$$\underset{k1\;:\;kn}{\cup }=\;\underset{k1\;:\;kn}{\cup }-\;k_{x}$$

$$\underset{kx}{\cup }=\;\underset{kx}{\cup }+\;k_{x}\;$$

else if W_kx_ = W_ky_ = max { W_k1_: W_kn_ } and $\underset{kx}{\cup }\geq \;\underset{ky}{\cup }\;$ then

$$\underset{k1\;:\;kn}{\cup }=\;\underset{k1\;:\;kn}{\cup }-\;\underset{kx+ky}{\cup }\;$$

$$\underset{kx}{\cup }=\;\underset{kx}{\cup }+\;k_{x}\;$$

End if

$${\text{W}}_{\underset{kx}{\cup }\;}\geq 90\%$$

End if

$$\underset{k1\;:\;kn}{\cup }=\;\text{Ø}$$

Return

$$\mathrm{KFCG}=\;\underset{kx}{\cup }\;$$

j represent genes in FRS; k represent the components, which is corresponding to genes in FRS; k_n_ represent the nth component; U represent unite; U_k_ represent unite of component responding to genes in FRS; U_kn_ represent the targets unite of k_n_; W represent the coverage of targets in U_j_.

### Calculation of the Potential Effect Score of Components

To quantify the comprehensive function of topological affect and control affection of KFCGs in the C-T-P network, we designed a component potential effect score calculation model. Usually, higher degree of components represents greater influence. The higher degree of the neighbor nodes of a component represents the higher control affection of this component in the topology of the network. It means that a component has a stronger control affection in the network when this component has much more targets and signaling pathways. Based on the above properties, we designed a component potential effect score calculation model, which was considered with both the network topology importance of KFCGs and the functional control ability of KFCGs. The specific model is as follows:

$$\mathrm{PES}=\;\sqrt[{2}]{\left(\sqrt{\text{Σ}\left(k_{n}\right)}\right)\;\times \;\left[\frac{\left|\;V\;\left(\;k_{\left(p\right)}\right)\;\right|}{\left|\;V\;\right|}\;\times \;\frac{\sum_{w\in k\left(p\right)}\left(\;\Delta_{k\left(p\right)}+1-dist_{\left(p,w\right)}\right)}{\max\;\left\{dist_{\left(p,w\right)}:w\;\in k\left(p\right)\right\}}\right]}$$

We defined the 56 components of KFCG and its 585 targets as node n. Then, the neighborhood of a node n is the set of nodes sharing an edge with n. The connectivity of node n, *k*_*n*_, is the size of its neighborhood. The degree of node n is the number of edges reaching n, which is equivalent to *k*_*n*_. *k*_(*p*)_ represents the component *k* of KFCG which can reach the pathway *p*. The relationship between *k* and pathway *p* contains two cases that component *k* effect the pathway *p* through its directive targets and through the proteins which can interact with its targets indirectly. V represents the collection of nodes within the network. |V| represents the number of nodes. $\Delta_{k\left(p\right)}$ is the maximum distance between component *k* and other genes passing through pathway *p*. *dist*_(*p*,__*w*)_ represents the length of a shortest path between pathway *p* and component *w*. The *dist*_(*p*,__*w*)_ is equal to infinite if C_(*p*)_ ≠ C_(*w*)_ *,* and it makes methods of this category cannot be applied to networks with disconnected genes. Finally, PESs were normalized into 0–1.

### Docking Simulations

Computer-simulated modeling can contribute to the prediction in the likelihood of molecular interactions. We obtained the 3D conformer of XXMD KFCG from ZINC (https://zinc.docking.org /) and PubChem (https://pubchem.ncbi.nlm.nih.gov /) and obtained proteins coded by genes involved in the comprehensive pathways. The affinity method and pyMOL were conducted in docking simulations and graph creation, respectively.

### Gene Ontology and Pathway Analysis

For analyzing the main function of targets, the clusterProfiler package of R software was used to perform GO analysis (Yu et al., 2012) and Kyoto Encyclopedia of Genes and Genomes (KEGG) pathway enrichment analysis (Draghici et al., 2007). According to the reports in using clusterProfiler package, the cutoff of *p*-values < 0.01 has the higher acceptance, and was used in the following enrichments analysis (Yu et al., 2012). The ggplot2 package and the Pathview (Luo et al., 2017) of R software were used in the graph creation and gene annotation, respectively.

## Experimental Validation

### Materials

Fetal bovine serum (FBS) and RPMI-1640 were purchased from ThermoFisher Biochemical Products (Beijing) Co., Ltd. Hypoxic bags were purchased from Mitsubishi Gas Chemical Company, Inc. (Japanese). Ferulic acid (≥98% purity by HPLC), zingerone (≥98% purity by HPLC), and vanillic acid (≥97% purity by HPLC) were purchased from Jiangsu Yongjian Pharmaceutical Technology Co., Ltd. (Jiangsu, China). Caryophyllene oxide (≥99% purity by HPLC) and methylephedrine hydrochloride (≥98% purity by HPLC) were purchased from TargetMol (United States) and Shenzhen Polymeri Biochemical Technology Co., Ltd. (Shenzhen, China), respectively. Edaravone (≥99% purity by HPLC) was purchased from TargetMol (United States). Cell Counting Kit-8 (CCK-8) was purchased from Dojindo Laboratories (Japanese).

### Cell Culture

The PC12 cell line was obtained from CHI SCIENTIFIC (Shanghai, China) and cultured in RPMI-1640 with FBS, penicillin 100 U/mL, streptomycin 100 μg/ml, respectively, at 37°C in a fully humidified 5% CO_2_ atmosphere.

### Oxygen and Glucose Deprivation Protocol

Oxygen and glucose deprivation (OGD) is a well-established *in vitro* model in studying the pathology and pharmacology of ischemic damage (Gu et al., 2013; Guo et al., 2013; Morán et al., 2017; Tian et al., 2020). Considering the actual clinical situations, patients usually take medications of XXMD in the poststroke (Pan et al., 2017; Zhong et al., 2020); we study the effect of components in treating stroke-based PC12 cells in the case that cells were protected with components after OGD. During the OGD, the cells were incubated in the culture medium without FBS in a hypoxic bag at 0.1% O_2_, 5% CO_2_, and 37°C for 18 h (Gu et al., 2013; Morán et al., 2017).

### Comparation of Effective Components and Non-Key Functional Components Group Components in PC12 Cells

To test the predictive power of our proposed model, the effective components of KFCGs (ferulic acid, zingerone, and vanillic acid) and two non-KFCG components (caryophyllene oxide and methylephedrine hydrochloride) were selected to validate our model *in vitro* experiments with PC12 cells. Edaravone that has been shown to reduce cell death was selected as a positive drug (Zang et al., 2018; Shou et al., 2019; Jiang et al., 2020). Effects of these components with different concentrations on cell viabilities were detected by CCK-8 assay.

CCK-8 assay was utilized to measure cell viability. Cells were seeded in 96-well plates (2 × 10^4^ per/well). After 24 h incubation, cells were treated without any components for 18 h in OGD. A control group without the treatments of OGD and components was taken at the same time. After the OGD period, cells were treated with 0.01, 0.1, 1, 10, 100, and 1,000 μM ferulic acid (Hassanzadeh et al., 2018; Moghadam et al., 2018; Nakayama et al., 2020), zingerone (Ho et al., 2013; Ruangsuriya et al., 2017), vanillic acid (Shen et al., 2018), caryophyllene oxide, methylephedrine hydrochloride, and 20 μM edaravone (Zang et al., 2018; Shou et al., 2019; Jiang et al., 2020), and were incubated with complete culture medium under normoxic condition for 18 h. Furthermore, a model group with OGD treatment and without components treatments was taken at the same time. Then, cells were changed to be cultured in a fresh complete culture medium with 10 μl of CCK-8 for a further 4 h. The absorbance was measured at 450 nm with a microplate reader (Infinite M200, TENAN, Switzerland). The experiments were repeated twice with three replicates each time.

### Statistical Analysis

All data were expressed as mean ± SEM. The differences between the model group and the control group were analyzed by Student t test. The differences between components treatments and model group were analyzed by one-way ANOVA for multiple comparisons. Results were considered statistically significant if the *p*-value was <0.05.

## Results

**FIGURE 1:**
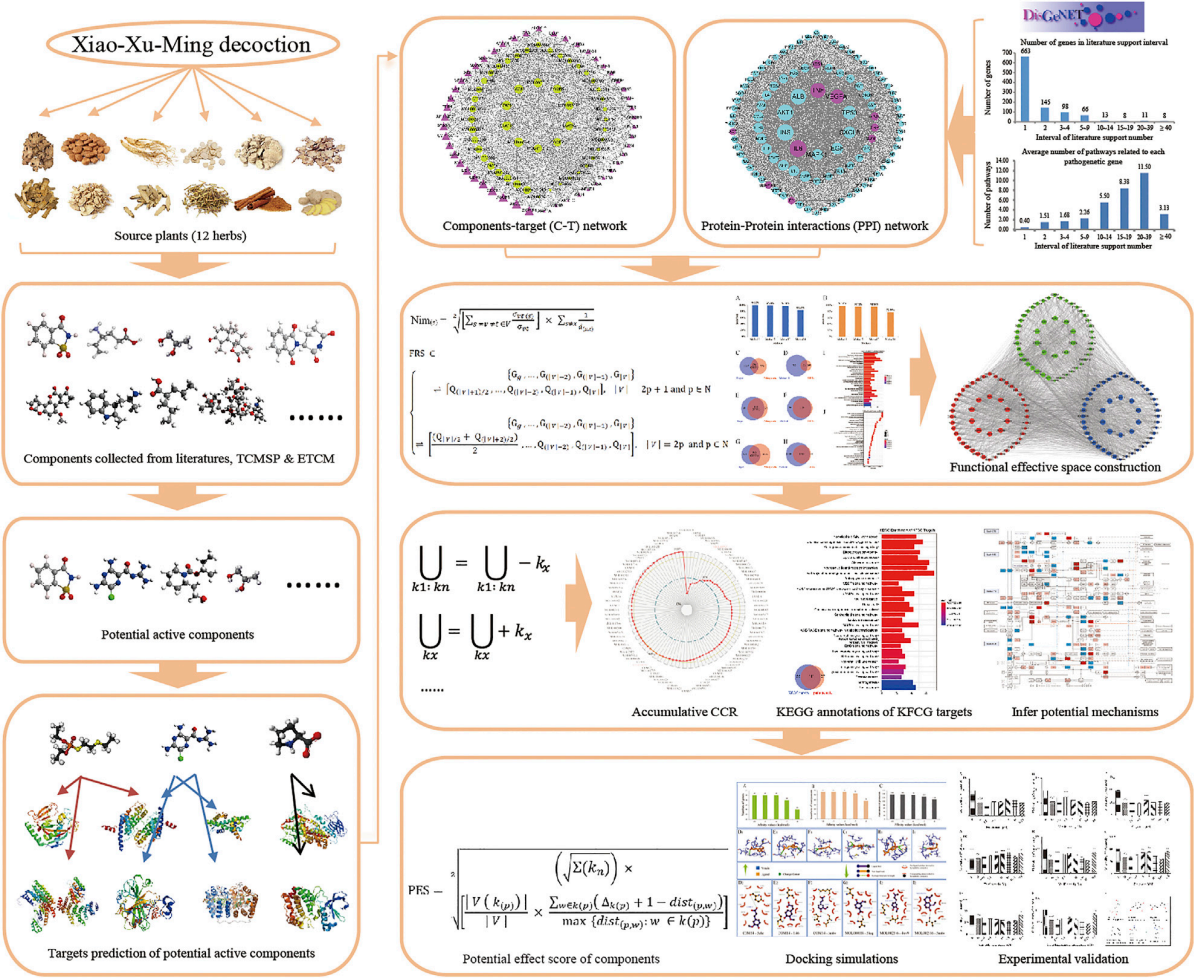
The work scheme of network pharmacology approach.

In this report, a new novel network pharmacology module was designed to detect the KFCG and elucidate the potential mechanism of XXMD in the treatment of stroke (Figure 1). First, all XXMD components were collected from the database. Second, the potential active components were selected from the XXMD components based on the proposed ADME-related models, and the targets of these potential active components were predicted by three published prediction tools. Third, the weighted gene regulatory network and the active component target network were used to construct FRS for determining the effective proteins. The effective proteins were used to select the KFCG based on the CCR model. Fourth, docking simulations were conducted based on KFCG and comprehensive pathway. Fifth, the KFCG and docking simulations were used to infer underlying molecular mechanism of XXMD in treating stroke. Finally, three components of KFCG, ferulic acid, zingerone, and vanillic acid, with highest PESs were validated by *in vitro* experiments with PC12 and HT22 cells.

### Identification of Pathogenetic Genes

The process of stroke is related to a series of phenotypic changes accompanied by alterations of genes expression. These genes may be labeled as pathogenetic genes at both the diagnostic and intervention levels. The collection and analysis of pathogenetic genes are the basis and key steps in understanding the pathogenetic genes of stroke and providing intervention strategies. To obtain more comprehensive pathogenetic genes, we extracted pathogenetic genes from the proved literatures in the DisGeNET database, related to both ischemic and hemorrhagic stroke (Supplementary Table S1).

**FIGURE 2:**
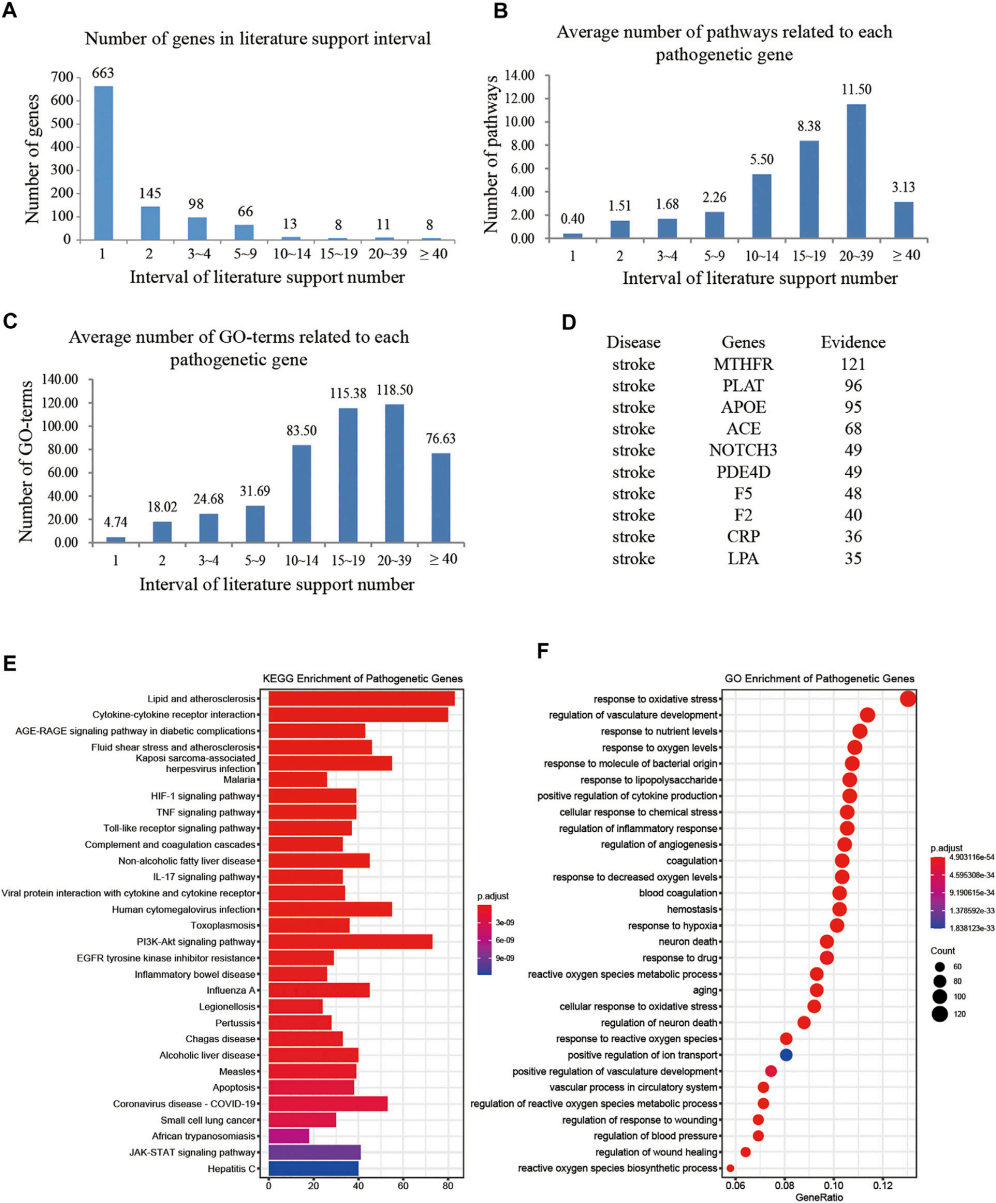
Study situations of pathogenetic genes. **(A)** Number of genes changes with the intervals of literature support; **(B)** Average number of signaling pathways changes with the intervals of literature support; **(C)** Average number of GO terms changes with the intervals of literature support; **(D)** the top 10 genes valued by the number of literatures support; **(E)** KEGG enrichment analysis of pathogenetic genes; **(F)** GO enrichment analysis of pathogenetic genes.

In total, 2,788 literatures provided conclusive evidence for further construction of intervention space, and in total 1,012 predicted target genes were retained as pathogenetic genes. All 1,012 genes were supported by at least one published report. Among these, 27 genes were supported by more than 15 published reports (Figure 2A).

To determine whether pathogenetic genes reported with higher number of evidence have more comprehensive function, we preformed KEGG and GO analysis, and got 138 signaling pathways and 2,802 GO terms (Figures 2E,F). The results showed that genes with higher number of evidence have more relation of signaling pathways and GO terms.

The genes supported with 20–39 evidences have the largest average number of signaling pathways and GO terms (Figures 2B,C). The top 10 genes supported with the largest number of evidence are MTHFR, PLAT, APOE, ACE, NOTCH3, PDE4D, F5, F2, CRP, and LPA (Figure 2D) and mainly associated with the lipid metabolism and cell activity of stroke. Methylene tetrahydrofolate reductase (MTHFR) and Apolipoprotein E (APOE) reduce the risk of atherosclerosis (AS) and stroke (Guo et al., 2019; Xie Q. et al., 2020; Ji et al., 2020; Mazdeh et al., 2020; Qin et al., 2020); angiotensin-converting enzyme (ACE) affects the renin-angiotensin system (RAS), regulates blood pressure, and affects the occurrence of ischemic stroke (IS) (Dušanović Pjević et al., 2019; Isordia-Salas et al., 2019); NOTCH3 affects the maturation and homeostasis of vascular smooth muscle cells, and causes cerebral ischemic events (Mukai et al., 2020; Young et al., 2020); phosphodiesterase 4D (PDE4D) promotes cyclic adenosine monophosphate (Liu et al.) degradation and apoptosis (Zhou et al., 2019) and increases the probability of stroke occurrence (Yue et al., 2019) and IS reperfusion injury (Lu et al., 2020).

Among the top 30 pathways of pathogenetic genes (Figure 2E), more than 10 pathways are related to stroke. For example, the pathway Cytokine-cytokine receptor interaction is related to cerebral ischemia-reperfusion injury (Wang et al., 2017) and is one of the important active pathways after stroke (Li L. et al., 2020); the HIF-1 signaling pathway is related to the neuromodulation (Xu H. et al., 2019; Yang W. et al., 2020), cellular activity (Sugumaran et al., 2020), ischemic angiogenesis (Liu Y. et al., 2019), and inflammation (Liu R. et al., 2019; Du et al., 2020). Furthermore, ischemic stroke has 14 similar pathogenetic genes, ACVRL1, APP, ABCC6, CST3, ENG, F7, JAK2, PLAT, PLAU, VHL, SH2B3, PDCD10, KIF1B, and CCM2, and has 48 similar signaling pathways to hemorrhagic stroke, whereas the stroke-related ID C0553692 is related to hemorrhagic stroke and to 29 pathogenetic genes and 48 signaling pathways. It indicated that ischemic stroke and hemorrhagic stroke are both important in the study of XXMD (Zhang et al., 2021). In addition, most of the top 30 GO terms of pathogenetic genes are closely associated with stroke, such as response to oxidative stress (GO:0006979) (Chen et al., 2020; Zhang et al., 2020d) and regulation of inflammatory response (GO:0050727) (Zhang et al., 2014; Xu S. et al., 2020).

### Construct Weighted Gene Regulatory Network of Stroke

The weighted gene regulatory network can contribute to understanding the pathogenetic genes and provide intervention strategies of stroke. To construct a weighted gene regulatory network, the comprehensive PPI network was combined from CMGRN and PTHGRN (Guan et al., 2014a; Guan et al., 2014b).

**FIGURE 3:**
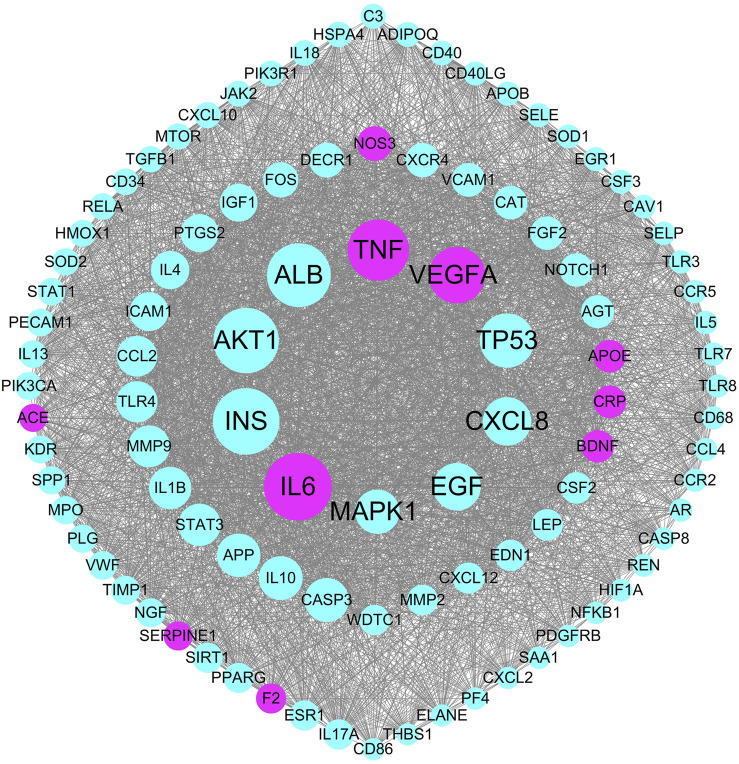
The top 100 genes in the weighted gene regulatory network of stroke. Node size represents weight of genes in the PPI network; red nodes represent the pathogenetic genes with more than 25 literature supports.

The pathogenetic genes were mapped to the PPI network to construct the weighted gene regulatory network of stroke. The network contains 949 nodes and 42,716 edges (Figure 3). To validate the reliability of the weighted gene regulatory network, we compared the consistency of the degree of nodes and weight of the nodes, and found that IL6, TNF, and VEGFA have the highest degrees with 374, 336, and 308, respectively, whereas the numbers of literatures supporting these genes are 34, 32, and 23, respectively. Numbers of literatures supporting NOS3, APOE, CRP, BDNF, and F2 are 31, 95, 36, 28, and 40, respectively. These genes also have large degrees in the network as 180, 167, 167, 166, and 152 edges, respectively, which are higher than the average degrees of all nodes in the network of 45.01. These results indicated that the weighted gene regulatory network is reliable for further analysis. Additionally, according to published reports, these genes are widely enriched in the pathways that are closely associated with stroke, such as Cytokine-cytokine receptor interaction (hsa04060) (IL6, TNF), PI3K-Akt signaling pathway (hsa04151) (IL6, NOS3, VEGFA), MAPK signaling pathway (hsa04010) (TNF, VEGFA), and Ras signaling pathway (hsa04014) (VEGFA). These results suggested that the weight gene regulatory network and weighted genes could reflect the pathogenetic genes of stroke, which also provided a reliable reference for the next step to construct the FRS.

### Components of Herbs in Xiao-Xu-Ming Decoction

By a systematic search for components from public databases, we obtained 1,490 components from 12 herbs in XXMD, including BS, CX, FF, FJ, FZ, GC, HQ, KXR, MH, RS, RG, and SJ (Supplementary Table S2). Meanwhile, we obtained additional 114 components from the 12 herbs according to literatures (Table 1).

### Select Potential Active Components

**FIGURE 4:**
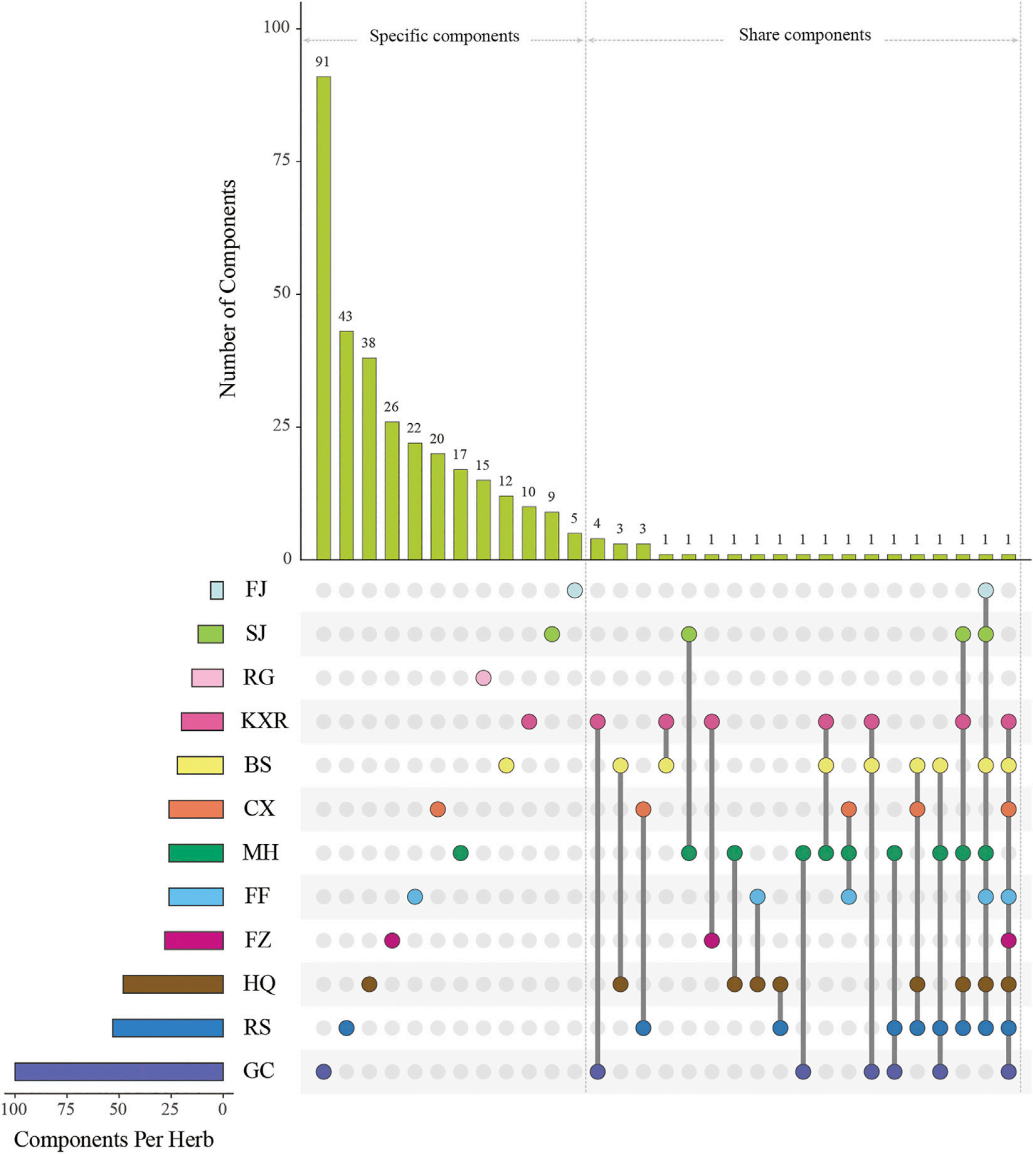
Components’ intersections of herbs in XXMD. “Components Per Herb” means the number of components of per herb; “Components’ intersections” means the unique components of each herb or the shared components of one herb and other herbs; FJ, SJ, RG, KXR, BS, CX, MH, FF, FZ, HQ, RS, and GC represent Stephania tetrandra S.Moore, Zingiber officinale Roscoe, Cinnamomum cassia (L.) J.Presl, Amygdalus communis L., Paeonia lactiflora Pall., Ligusticum striatum DC., Ephedra alata Decne., Saposhnikovia divaricata (Turcz.) Schischk., Aconitum wilsonii Stapf ex Veitch, Scutellaria baicalensis Georgi, Ginseng quinquefolium (L.) Alph.Wood and Glycyrrhiza uralensis Fisch, respectively.

We obtained 220 potential active components from these 1,490 components based on three ADME-related models including OB, Caco-2, and DL (Supplementary Table S3). Except ADME prediction, experimental chemical analysis also plays important roles in the study of substances basis and mechanism of herbs in the formulas. Thus, we regarded the additional 114 components collected from literatures with these 220 ADME-predicted components as potential active components of XXMD (Figure Figure4; table 2, Supplementary Table S4).

**TABLE 2:** Statistics on the number of XXMD components collected in the databases and literatures.

Herbs	Components from databases	ADME-predicted components from databases	ADME-predicted and literatures-selected components
BS	85	9	22
CX	189	6	26
FF	173	20	26
FJ	50	4	6
FZ	65	16	28
GC	280	89	100
HQ	143	36	48
KXZ	113	18	20
MH	364	21	26
RG	100	11	15
RS	200	22	53
SJ	275	5	12
Total	1,490	220	334

### Shared Components of Herbs in Xiao-Xu-Ming Decoction

A total of nine components are contained in three or more than three herbs (Supplementary Table S5). These components are sitosterol (MOL000359), beta-sitosterol (MOL000358), stigmasterol (MOL000449), gallic acid (COM4), kaempferol (MOL000422), quercetin (MOL000098), mairin (MOL000211), (+)-catechin (MOL000492), and mandenol (MOL001494). Among them, beta-sitosterol is present in MH, BS, RS, FF, HQ, FJ, and SJ, and can exert anti-inflammatory (Navarro et al., 2001) and antioxidant efficacies (Cao et al., 2018; Yuan et al., 2019; Devaraj et al., 2020). It has been proved to be an important component in the prescriptions of Hua-Feng-Dan and Buyang Huanwu decoction in the treatment of stroke (Yang P. et al., 2020; Gao et al., 2021). Stigmasterol is present in KXR, MH, RS, HQ, and SJ, which can promote cholesterol secretion and reduce AS (Lifsey et al., 2020); (+)-catechind is present in KXR, MH, and BS and can regulate the expression of proteins p-Akt and p-GSK-3b (Wang J. et al., 2020) which can inhibit the production of cell toxicity when PC12 cells are under hypoxic conditions (Zhou and Li, 2020). Kaempferol is present in MH, GC, BS, RS and can improve neurological deficits in cerebral ischemia/reperfusion (Zhai, 2019).

### Specific Components of Herbs in Xiao-Xu-Ming Decoction

Except the shared components, most of the herbs possess their unique components (Supplementary Table S6). For example, the numbers of unique components of FZ, GC, and RS are 26, 91, and 43, respectively. In these herbs, some unique ingredients have a special therapeutic effect on stroke. For example, luteolin (MOL000006) is an unique component in MH, has properties of anti-inflammatory, neuroprotective, anti-allergic, and vascular protection (Qiu et al., 2013; Pandurangan and Esa, 2014; Su et al., 2021), and has been proved to enhance mitochondrial function by increasing the transduction of Sirtuin 3 (SIRT3) through the SIRT3/AMPK/mTOR pathway and to reduce the infarcted area of middle cerebral artery occlusion (MCAO) rat model (Liu S. et al., 2020). It also reduces oxidative damage to cells (Zhang Z. et al., 2019) and enhances cell viability and downregulates apoptosis (Luo S. et al., 2019). Skullcapflavone II (MOL002927) is a unique component in HQ and has properties of anti-inflammatory (Jang et al., 2012). It inhibits the proliferation of tumor cells (Tayarani-Najarani et al., 2012) and cancer cells (Bonham et al., 2005) and maintains the integrity of extracellular matrix (Lee et al., 2019). Hesperetin (MOL002341) is a unique component in FJ and regulates lipid metabolism (Xiong et al., 2019), lowers blood pressure, and prevents endothelial dysfunction (Yamamoto et al., 2008; Morand et al., 2011). Therefore, these components can be considered curative elements in treating stroke.

### Construction of Component-Target Network

**FIGURE 5:**
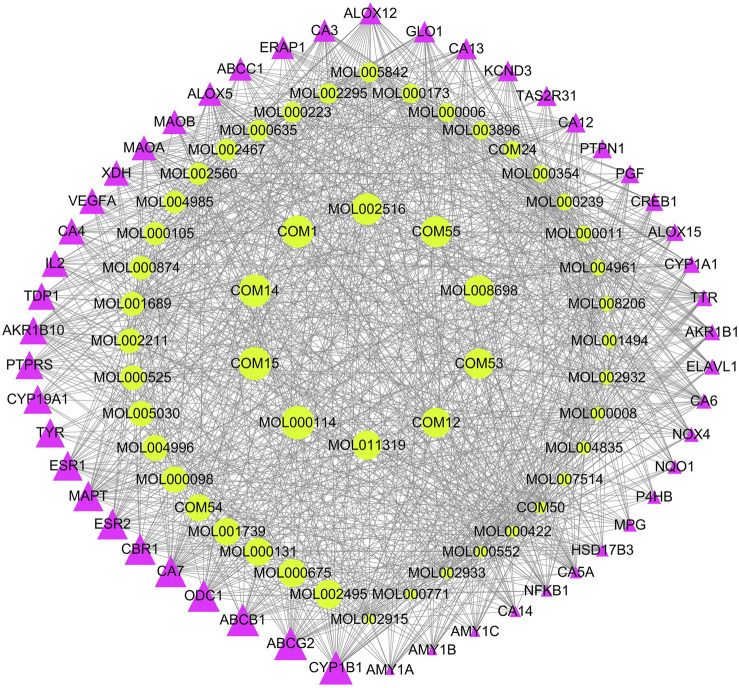
The top 50 components and top 50 targets in the C-T network. Green represents the components of XXMD, Red represents targets; the larger of the dot represents the greater weight of the components or targets.

To explore the potential mechanism of XXMD in the treatment of stroke, 334 potential active components and their 1,329 targets (Supplementary Table S7) are used in constructing the component-target (C-T) network. The top 50 components and top 50 targets valued by degree in the C-T network were shown in Figure 5. Several of these potential active components are related to multiple targets, resulting in 12,395 C-T associations between 334 active components and 1,329 targets. The average number of targets per component is 4.02, indicating that there are multi-targets characteristics of XXMD in the treatment of stroke. Among these components, vanillic acid (MOL000114) has the highest number of targets as 258, followed by components of L-tryptophan (COM15), ferulic acid (COM14), benzoic acid (COM1), and zingerone (MOL002516), etc. Most of these components are related to inflammation and neuroprotection in stroke. For example, dihydrocapsaicin is a kind of capsaicin compound which can mediate the generation and functional recovery of blood vessels after ischemic stroke (IS) (Wu et al., 2019; Janyou et al., 2020); quercetin is a flavonoid that protects the neurons of IS (Wang et al., 2018; Zhao et al., 2018). These results identified a range of plausible biological responses to XXMD, and demonstrated that the XXMD might work in a multi-target manner in the treatment of stroke.

### Effective Proteins Selection and Validation From Functional Response Space

Here, we constructed a disease-targets network based on the weighted gene regulatory network and C-T network. This network contains 1,836 nodes and 25,383 edges (Supplementary Table S8).

To evaluate the effectiveness of the FRS, we defined the intersection of pathogenetic genes and XXMD targets as the un-optimized effective targets (UETs) of XXMD and defined the genes that were included in the FRS as effective proteins. Then we evaluated the effective proteins with three evaluation indicators: 1). the proportion of effective proteins in the number of UETs; 2). the proportion of effective proteins in the number of UETs enriched pathways; 3). the proportion of effective proteins in the number of UETs enriched GO-terms.

**FIGURE 6:**
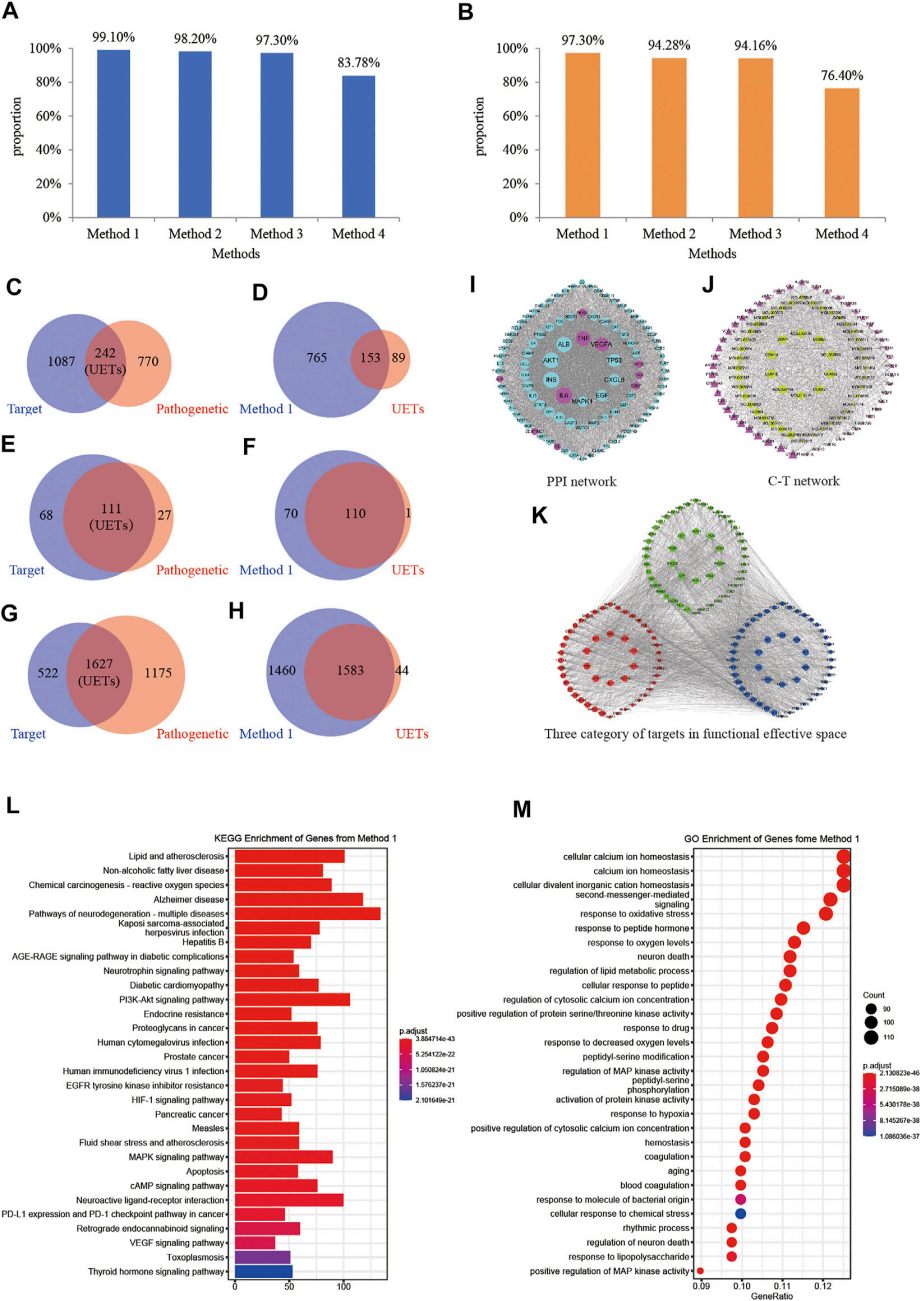
Construction and validation of functional response space. **(A)** Proportions of the signaling pathways of genes which were selected by different methods to the signaling pathways of the UETs. Method 1, method 2, method 3, and method 4 in **(A,B)** represent our proposed method, the traditional model Degree, ClosenessCentralty, and ClusteringCoefficient, respectively. **(B)** Proportions of the GO terms of genes which were selected by different methods to the GO terms of the UETs. **(C)** The number of pathogenetic genes and XXMD targets, respectively; **(D)** The number of the genes in FRS and UETs, respectively; **(E)** The number of signaling pathways of XXMD targets and pathogenetic genes, respectively; **(F)** The number of signaling pathways of the genes in FRS and UETs, respectively; **(G)** The number of GO terms of XXMD targets and pathogenetic genes, respectively; **(H)** The number of GO terms of the genes in FRS and UETs, respectively; **(I)** PPI network; **(J)** C-T network; **(K)** The top 200 genes of FRS are shown in the figure and include three categories of targets. Blue nodes represent disease-specific targets, red nodes represent essential common targets, green nodes represent component-specific targets. **(L)** KEGG enrichment analyses of the genes in FRS; **(M)** GO terms of the genes in FRS.

We used four methods in the optimization of targets and pathogenetic genes. Among these four methods, the best optimal method was method 1, our proposed node importance calculation method (Figures 6D,F,H). Based on our proposed model, 918 nodes were filtrated as important nodes, and also defined as the effective proteins. The effective proteins and their interactions were used to construct the FRS. There were three subtypes of effective proteins in the FRS (Figure 6K). The first subtype represents essential common targets, which directly linked pathogenetic genes and herbs targets. The second subtype represents disease-specific targets. The third subtype represents component-specific targets. These three subtypes indicated that the effective proteins could represent the effect of pathogenetic genes and XXMD targets.

The numbers of effective protein-enriched pathways and GO-terms were 180 and 3,043, respectively (Figures 6L,M). Before the optimization, the numbers of XXMD targets and pathogenetic genes of stroke were 1,329 and 1,012, respectively (Figure 6C), the numbers of targets and pathogenetic genes enriched pathways were 179 and 138, respectively (Figure 6E), whereas the numbers of targets and pathogenetic genes enriched GO-terms were 2,149 and 2,802, respectively (Figure 6G). The enriched pathways of effective proteins accounted for 99.10% of UETs enriched pathways (Figure 6F). The GO terms accounted for 97.30% of UETs enriched GO Terms (Figure 6H). These results confirmed the reliability and accuracy of our approach in constructing FRS and further demonstrated that the effective proteins in the FRS played a leading role in the pathogenetic genes of stroke.

KEGG and GO enrichment analysis of the effective proteins in FRS showed that the effective proteins could play a role in treating stroke. Among the 180 signaling pathways of the effective proteins (Figure 6L), PI3K-Akt signaling pathway could increase the expression of anti-apoptotic protein (Bcl-2, Bcl-XL), inhibit the expression of apoptosis protein (caspase-3, Bax), and could reduce neuronal apoptosis during stroke or hypoxia and hypoglycemia reoxygenation (OGD/R) (Wu et al., 2019; Xie W. et al., 2020; Miao et al., 2020); Neuroactive ligand-receptor interaction could affect expressions of GRM5, GRIK1, GRIK3, GABRA3, ADRA2C, VIPR2 and other genes (Zhang S. et al., 2019), affecting the ability of nerve transmission in inhibiting stroke occurrence (Riccio et al., 2012; Takenouchi et al., 2014) and affecting the pro-inflammatory cytokine releasement (Olson et al., 2015). Among the 3,043 GO terms of the effective proteins (Figure 6M), some of these GO terms were closely associated with the occurrence and recovery of stroke, such as response to oxidative stress (GO:0006979) (Chen et al., 2020; Zhang et al., 2020d), regulation of lipid metabolic process (GO:0019216) (Mahrooz et al., 2019; Sharma et al., 2020), regulation of inflammatory response (GO:0050727) (Xu S. et al., 2020; Du et al., 2020), regulation of blood circulation (GO:1903522) (Du et al., 2020; Gutierrez-Vargas and Cardona-Gomez, 2020), and neuron death (GO:0070997) (Iadecola and Anrather, 2011).

### Key Functional Components Group Selection and Validation

**FIGURE 7:**
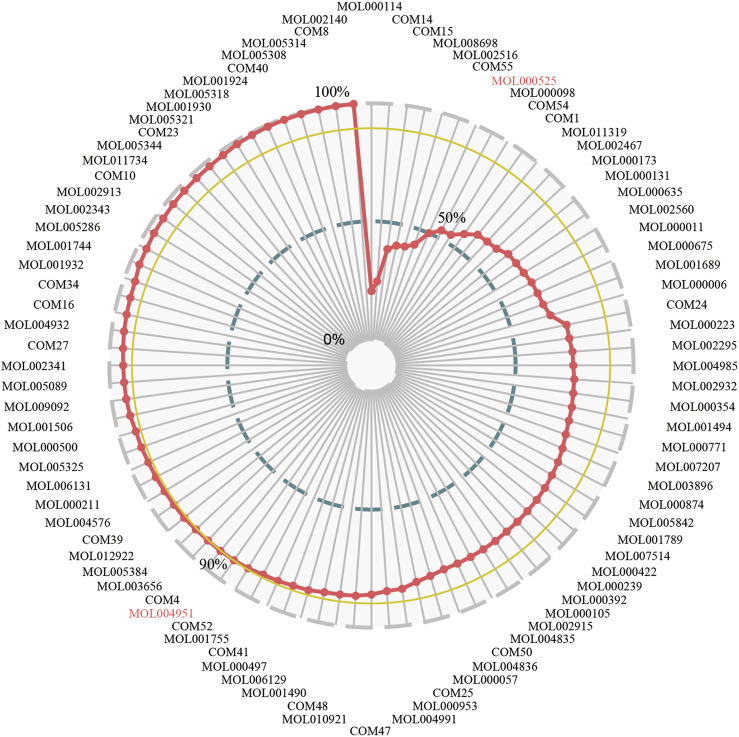
The contribution ratio of potential active components and the definition of KFCG in XXMD. MOL and COM ids represent the potential active components; the red line represents that the contribution ratio of potential active components values from 0 to 100%; the blue line represents the contribution ratio of 50%; the yellow line represents the contribution ratio of 90%; 56 components have the contribution ratio of 90% and were defined as the KFCG of XXMD.

The CCR model was established to optimize the effective components and get the KFCG that could be used to illustrate the potential molecular mechanism of XXMD in the treatment of stroke. According to the contribution accumulation score ranking, targets of the top seven components including vanillic acid, ferulic acid, L-tryptophan, Dihydrocapsaicin, zingerone, rhamnetin, and Norwogonin could cover 50.00% of effective proteins. For further analysis, targets of the top 56 components could cover 90.00% of effective proteins, and these 56 components were selected as KFCG (Figure 7; Table 3, Supplementary Figure S1). The high targets coverage of effective proteins of 90.00% proved that the KFCG of XXMD might play the leading role and generate combination effects in the therapy of stroke.

**TABLE 3:** Information of 56 components in KFCG.

ID	Molecule name	ob	caco2	DL	ID	Molecule name	ob	caco2	DL
MOL000006	Luteolin	36.16	0.19	0.25	MOL002560	Chrysin	22.61	0.70	0.18
MOL000011	(2R,3R)-3-(4-hydroxy-3-methoxy-phenyl)-5-methoxy-2-methylol-2,3-dihydropyrano[5,6-h] [1,4]benzodioxin-9-one	68.83	0.21	0.66	MOL002915	Salvigenin	49.07	0.86	0.33
MOL000057	DIBP	49.63	0.85	0.13	MOL002932	Panicolin	76.26	0.84	0.29
MOL000098	Quercetin	46.43	0.05	0.28	MOL003896	7-Methoxy-2-methyl isoflavone	42.56	1.16	0.20
MOL000105	Protocatechuic acid	25.37	0.10	0.04	MOL004835	Glypallichalcone	61.60	0.76	0.19
MOL000114	Vanillic acid	35.47	0.43	0.04	MOL004836	Echinatin	66.58	0.38	0.17
MOL000131	EIC	41.90	1.16	0.14	MOL004951	Isoliquiritin	8.61	−1.36	0.60
MOL000173	Wogonin	30.68	0.79	0.23	MOL004985	Icos-5-enoic acid	30.70	1.22	0.20
MOL000223	Caffeic acid	25.76	0.21	0.05	MOL004991	7-Acetoxy-2-methylisoflavone	38.92	0.74	0.26
MOL000239	Jaranol	50.83	0.61	0.29	MOL005842	Pectolinarigenin	41.17	0.70	0.30
MOL000354	Isorhamnetin	49.60	0.31	0.31	MOL006129	6-methylgingediacetate2	48.73	0.55	0.32
MOL000392	Formononetin	69.67	0.78	0.21	MOL007207	Machiline	79.64	0.78	0.24
MOL000422	Kaempferol	41.88	0.26	0.24	MOL007514	Methyl icosa-11,14-dienoate	39.67	1.47	0.23
MOL000497	Licochalcone a	40.79	0.82	0.29	MOL008698	Dihydrocapsaicin	47.07	0.98	0.19
MOL000525	Norwogonin	39.40	0.60	0.21	MOL010921	Estrone	53.56	1.01	0.32
MOL000635	Vanillin	52.00	0.68	0.03	MOL011319	Truflex OBP	43.74	0.90	0.24
MOL000675	Oleic acid	33.13	1.17	0.14	COM1	Benzoic acid	—	—	—
MOL000771	p-coumaric acid	43.29	0.46	0.04	COM14	Ferulic acid	—	—	—
MOL000874	Paeonol	28.79	0.93	0.04	COM15	L-tryptophan	—	—	—
MOL000953	CLR	37.87	1.43	0.68	COM24	Guanosine	—	—	—
MOL001490	bis [(2S)-2-ethylhexyl] benzene-1,2-dicarboxylate	43.59	0.98	0.35	COM25	Uridine	—	—	—
MOL001494	Mandenol	42.00	1.46	0.19	COM41	Caffeine	—	—	—
MOL001689	Acacetin	34.97	0.67	0.24	COM47	Harmine	—	—	—
MOL001755	24-Ethylcholest-4-en-3-one	36.08	1.46	0.76	COM48	Hyperoside	—	—	—
MOL001789	Isoliquiritigenin	85.32	0.44	0.15	COM50	Rhamnetin	—	—	—
MOL002295	Cinnamic acid	19.68	0.91	0.03	COM52	Yohimbine	—	—	—
MOL002467	6-gingerol	35.64	0.54	0.16	COM54	10-gingerol	—	—	—
MOL002516	Zingerone	25.23	0.87	0.05	COM55	10-shogaol	—	—	—

### Kyoto Encyclopedia of Genes and Genomes Enrichment Analysis of Key Functional Components Group Targets

**FIGURE 8:**
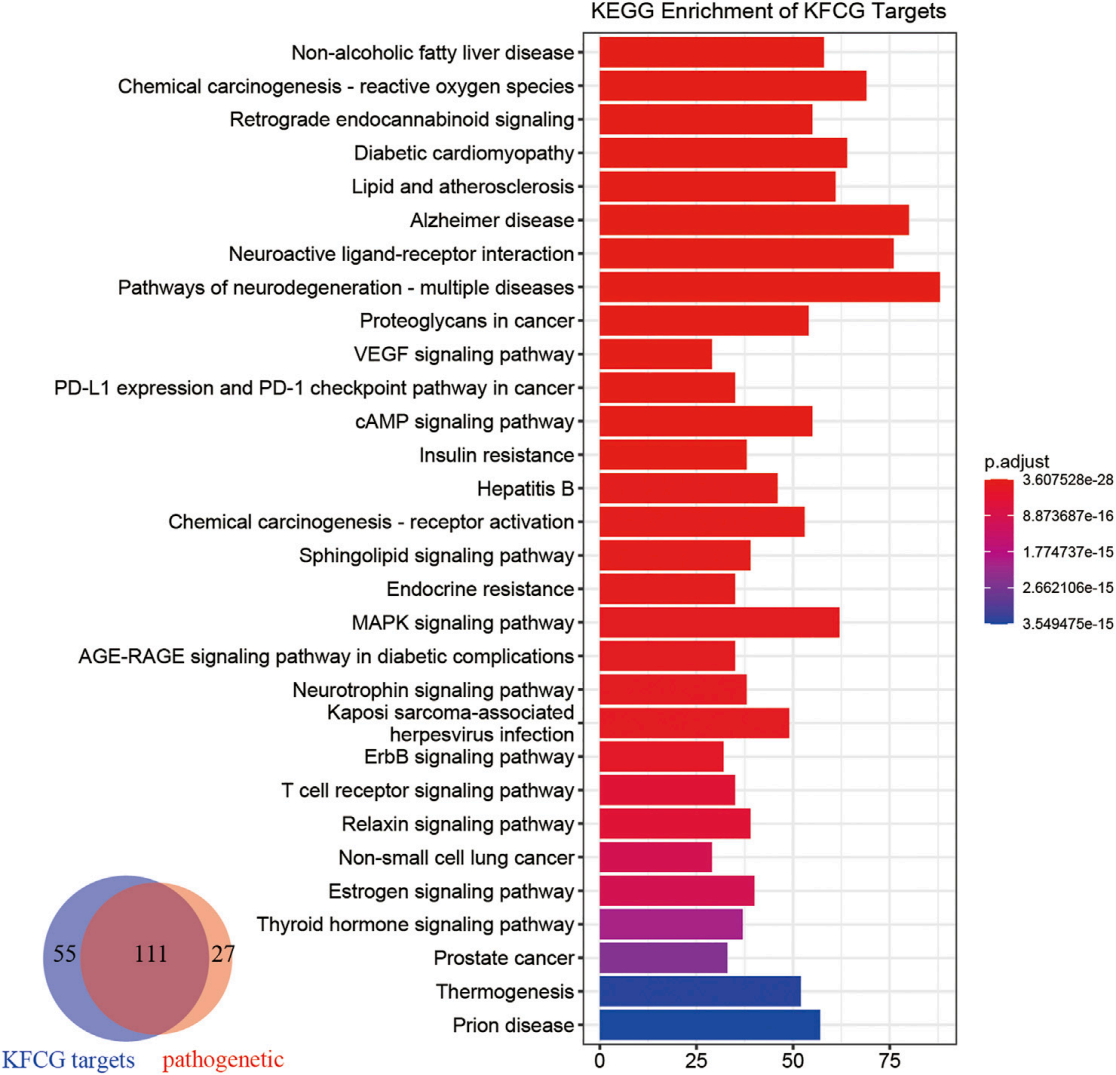
Pathway enrichment analyses of the targets of KFCG. The color change represents the significance of the enrichment of genes.

To analyze XXMD in the treatment of stroke at the functional level, we performed pathway analysis using KFCG targets. The number of KFCG targets enriched pathways is 166 which can cover 80.43% of pathogenetic genes enriched pathways (Figure 8).

These KFCG targets were involved in 166 pathways such as Retrograde endocannabinoid signaling (hsa04723), neuroactive ligand-receptor interaction (hsa04080), and cAMP signaling pathway (hsa04024). Most of these signaling pathways have been reported to be related to the occurrence of stroke or the recovery in poststroke. For example, neuroactive ligand–receptor interaction can affect the expression of genes such as GRM5, GRIK1, GRIK3, GABRA3, ADRA2C, and VIPR2 (Zhang S. et al., 2019), thereby affecting the ability of nerve transmission (Riccio et al., 2012; Takenouchi et al., 2014) and the releasing of pro-inflammatory cytokines (Olson et al., 2015); the cAMP signaling pathway can regulate the expression of Adora2a, Drd2, and Pde10a and the expression of DEG-targeted transcription factors (TFs), such as androgen receptor system (AR), contributing to the recovery of brain (Ito et al., 2018); PI3K-Akt signaling pathway (hsa04151) can increase the expression of anti-apoptotic proteins (Bcl-2, Bcl-XL), inhibit the expression of apoptotic protein (caspase-3, Bax), and reduce the neuronal apoptosis (Xie W. et al., 2020; Miao et al., 2020; Wu et al., 2020).

Stroke is classified into hemorrhagic stroke and IS, and has the characteristics of multisystem cross-effects, mainly involving the cerebrovascular system and nervous system. Studies have shown that the occurrence of stroke is related to cerebral microvessels, neural information transmission, neuronal activity (Wu et al., 2020), and inflammation (Olson et al., 2015). According to previous extensive studies, stroke has been confirmed to be associated with some pathways such as vascular endothelial growth factor (VEGF), signaling pathway (hsa04370), cAMP signaling pathway, MAPK signaling pathway (hsa04010), and PI3K-Akt signaling pathway. In our study, the numbers of KFCG genes enriched in the above four pathways were 29, 55, 62, and 60, respectively. The reports showed that the VEGF signaling pathway could affect the protein activity of VEGF, mediate in angiogenesis and blood supply (Wu et al., 2019), reduce the formation of brain edema in the poststroke (Peng et al., 2014; Hu et al., 2019), and could promote dendrite and synaptic plasticity and improve nerve recovery in stroke (Shim and Madsen, 2018; Xie et al., 2019). The cAMP signaling pathway could regulate the activities of Adora2a, Drd2, and Pde10a, contributing to the recovery in poststroke (Ito et al., 2018). It could also regulate the activity of DEG-targeted transcription factors (TFs), such as the androgen receptor system (AR), contributing to the recovery of brain (Zhang H. et al., 2019). The PI3K-Akt signaling pathway could reduce neuronal apoptosis during stoke or OGD/R (Xie W. et al., 2020; Miao et al., 2020; Wu et al., 2020). The MAPK signaling pathway could inhibit the apoptosis of nerve cells (Han et al., 2013; Han et al., 2020), increase the activities of ERK, JNK, and p38 (Hong et al., 2020), reduce the activities of PLA2, IL1, TNF, IL1β, IL6, and IL8 (Dong et al., 2019; Ding Y. et al., 2020), and regulate the synthesis of proteins such as ASPK/JNK, p38, and P-SAPK/JNK (Zeng et al., 2019).

**FIGURE 9:**
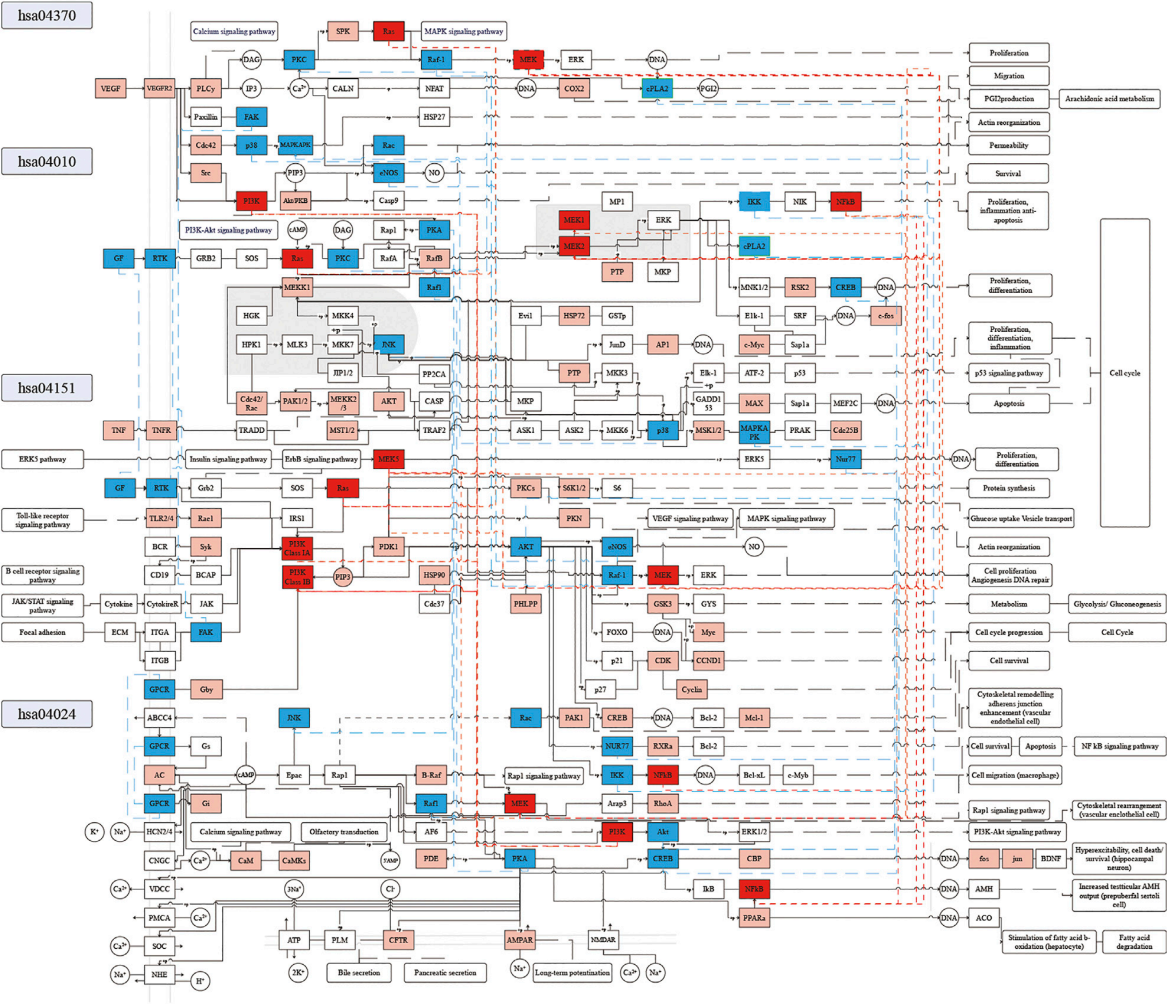
Distribution of targets of KFCG in the comprehensive pathway. Red, blue, and pink frames represent the KFCG targets or proteins enriched in 3, 2, and 1 pathways, respectively.

To further explore the potential mechanism of XXMD in the treatment of stroke, we constructed a comprehensive pathway with these four pathways, VEGF signaling pathway (hsa04370), MAPK signaling pathway (hsa04010), PI3K-Akt signaling pathway (hsa04151), and cAMP signaling pathway (hsa04024) (Figure 9).

Combining KFCG targets and these four pathways, we speculated the potential mechanism of XXMD to be two important aspects: 1) KFCG can benefit for cell proligeration, migration, survival, and DNA repair. 2) KFCG can regulate the activities of proteins which can interact between multiple pathways to play a role in the treatment of stroke by XXMD.

1) KFCG can benefit for cell survival and DNA repairation.

In the cAMP signaling pathway, KFCG can activate the activities of PI3K (genes PIK3CA, PIK3CB, PIK3CD, PIK3R1, PIK3R2, PIK3R3) (Xin et al., 2020) and Akt (genes AKT1, AKT2, AKT3), thereby regulating the gene expression and proliferation through the PI3K-Akt signaling pathway. For example, the deletion of PI3Kγ can upregulate 3′,5′-cAMP signaling in microglia, resulting in an increased release of microglial MMP-9 and a decrease in microglial phagocytic capacity (Schmidt et al., 2016). KFCG can phosphorylate Raf1 (gene RAF1), MEK (genes MAP2K1, MAP2K2) (Namura et al., 2001), CREB (genes CREB3, CREB1, CREB3L4, CREB3L2, CREB3L3, CREB3L1, CREB5) (Liu J. et al., 2019; Gao et al., 2020b; Queme et al., 2020), CBP (genes CREBBP, EP300), and can activate the activities of fos (gene FOS) and jun (gene JUN), thereby inhibiting the hyperexcitability and regulating cell death/survival of hippocampal neuron. For example, mitogen-active protein/extracellular signal-regulated kinase (MEK) inhibition can protect the hippocampus against forebrain ischemia (Namura et al., 2001). High phosphorylation of cAMP response element binding protein (CREB) is the target of 16 components of KFCG, including MOL000114, MOL000006, MOL000098, etc. (Supplementary Table S10). It can represent the high activity of CREB and can increase the expression of brain-derived neurotrophic factor (BDNF) which can enhance the synaptic efficiency and structural plasticity effectively as a most important neuronal protective factor and a prime mediator of synaptic plasticity (Leal et al., 2017; Lin et al., 2018). In addition, KFCG can regulate the activities of phosphorylate CFTR (gene CFTR) and AMPAR (genes GRIA1, GRIA2, GRIA3, GRIA4), which were located on the cell membrane, thereby affecting the transport of Ca^2+^, K^+^, and Na^+^ in cell membrane and affecting the process of pancreatic secretion, bile secretion, and cardiac muscle contraction.

In the VEGF signaling pathway, KFCG can regulate the activities of VEGF (gene VEGFA), VEGFR2 (gene KDR), PLC (genes PLCG1, PLCG2), and PKC (genes PRKCA, PRKCB, PRKCG), thereby affecting the calcium signaling pathway. KFCG can regulate the activities of PKC (genes PRKCA, PRKCB, PRKCG), Ras (genes HRAS, KRAS, NRAS) (Ding M.-H. et al., 2020), Raf-1 (gene RAF1) (Ding M.-H. et al., 2020), phosphorylate SPK (genes SPHK1, SPHK2), and MEK (genes MAP2K1, MAP2K2) (Ding M.-H. et al., 2020), thereby affecting the arachidonic acid metabolism and cell proliferation through the MAPK signaling pathway. The reports have shown that Ras and MEK can play actions in Ras/Raf/MEK/ERK signaling pathways and can be regulated by some miRNAs such as miRNA-21 and miRNA-26a, which can induce angiogenesis to aid in blood vessel formation for vascular tissue engineering in ischemic diseases (Ding M.-H. et al., 2020).

In the MAPK signaling pathway, KFCG can regulate the activities of Ras (genes RRAS2, MRAS, HRAS, KRAS, NRAS, RRAS), MEK1 (gene MAP2K1), MEK2 (gene MAP2K2) (Akgun-Dogan et al., 2019) and c-fos (gene FOS), and can regulate the activities of MEK5 (gene MAP2K5) and Nur77 (gene NR4A1) to regulate cell proliferation and differentiation. At the same time, the reports also show that germline mutations of RAS superfamily (KRAS, NRAS, HRAS, RRAS) and MAPK cascade (MEK1, MEK2) can cause strokes (Garavelli et al., 2015; Akgun-Dogan et al., 2019). Besides, KFCG can also phosphorylate IKK (genes CHUK, IKBKB, IKBKG) and regulate the activities of NFκB (genes NFKB1, NFKB2, RELA, RELB), thereby regulating cell Proliferation, inflammation, and anti-apoptosis. In addition, KFCG can also regulate the activities of TNF (gene TNF) (Ma et al., 2020; Meng et al., 2020), TNFR (gene TNFRSF1A), AKT (genes AKT1, AKT2, AKT3) (Gao et al., 2008), PTP (genes DUSP3, PTPN7, PTPRR), PTPN5), AP1 (genes FOS, JUN), MAX (gene MAX), and other protein activities, thereby affecting the cell cycle comprehensively. At the same time, the reports also have shown that the lower activities of proinflammatory factor TNF can benefit against IS (Ma et al., 2020).

In the PI3K-AKT signaling pathway, KFCG can regulate the activities of TLR2/4 (genes TLR2, TLR4) (Guo and Zhu, 2019), Rac1 (gene RAC1), PI3K Class IA (genes PIK3CA, PIK3CB, PIK3CD, PIK3R1, PIK3R2, PIK3R3), PI3K Class IB (genes PIK3R5, PIK3R6, PIK3CG) (Kong et al., 2016; Li J. et al., 2019), AKT (genes AKT1, AKT2, AKT3) (Chen et al., 2003) and can phosphorylate GSK3 (gene GSK3B) (Krafft et al., 2012), thereby promoting the neurorestorative activity in poststroke. Besides, KFCG can also regulate the activities of Ras (genes HRAS, KRAS, NRAS), Raf-1 (gene RAF1) and can phosphorylate MEK (genes MAP2KA, MAP2K2) (Bai et al., 2015), thereby affecting cell proliferation angiogenesis DNA repair through VEGF and MAPK signaling pathways. The reports have also shown that the inhibition of MEK can inhibit the activity of VEGF and be helpful for the decrease in the occurrence of stroke (Bai et al., 2015). KFCG can phosphorylate IKK (genes CHUK, IKBKB, IKBKG) and regulate the activities of NFκB (genes NFKB1, RELA), thereby promoting cell survival by the NFκB signaling pathway.

2) KFCG can regulate the activities of proteins which can interact between multiple pathways to play a role in the treatment of stroke by XXMD.

For example, in the 585 KFCG target enrichments, four proteins existed in three pathways and whose activities could be affected by KFCG, and were as MEK (pathways of hsa04024, hsa04370, and hsa04151), NFκB (hsa04024, hsa04010, hsa04151), PI3K (hsa04024, hsa04370, hsa04151), and Ras (hsa04370, hsa04010, hsa04151) (Figure 9). Meanwhile, 18 proteins existed in two pathways and whose activities could be affected by KFCG, such as Akt, cPLA2, and CREB (Figure 9). Most of these proteins have been proved to be closely related to stroke. NFκB and AKT1 can both affect inflammation and anti-apoptosis in PI3K-Akt, cAMP, and MAPK signaling pathway. RAF1 can affect the activity of Raf1 in the cAMP signaling pathway and the MAPK signaling pathway, thereby affecting hyperexcitability, cell death/survival, proliferation, and differentiation. In addition, we also found that KFCG could affect many other signaling pathways indirectly such as insulin signaling pathway, ErbB signaling pathway, calcium signaling pathway, Rap1 signaling pathway, and NFκB signaling pathway through the four pathways that were shown in the compressed stroke pathways (Figure 9).

The results of the compressed stroke pathways indicated that the KFCG were important and effective in the stroke treatment, and these effects could be achieved through a multichannel biological process. This result suggested that we need to consider the relationship of components and targets and need to consider the interactions between different pathways in the treatment of stroke.

### The Calculation of the Potential Effect Score of Components

Based on the component potential effect score calculation model we constructed above, we calculated the PESs of 56 KFCG (Table 4). These PESs both considered about the network topology importance and the functional control ability of KFCGs. In the following analysis, we selected the top three components, vanillic acid, ferulic acid, and zingerone, for subsequent validations to test the predictive power of the model.

**TABLE 4:** Potential effect score of 56 KFCGs.

ID	Molecule name	PESs	ID	Molecule name	PESs
MOL000114	Vanillic acid	1.00	MOL001494	Mandenol	0.39
COM14	Ferulic acid	0.95	MOL000422	kaempferol	0.39
MOL002516	Zingerone	0.78	MOL000239	Jaranol	0.38
COM55	10-shogaol	0.69	MOL005842	Pectolinarigenin	0.37
MOL008698	Dihydrocapsaicin	0.66	MOL000675	Oleic acid	0.36
COM15	L-tryptophan	0.65	COM1	Benzoic acid	0.36
MOL002467	6-gingerol	0.63	MOL000392	Formononetin	0.36
MOL000635	Vanillin	0.60	COM50	Rhamnetin	0.35
MOL000098	Quercetin	0.60	MOL003896	7-Methoxy-2-methyl isoflavone	0.33
MOL000006	Luteolin	0.59	MOL004985	Icos-5-enoic acid	0.31
COM54	10-gingerol	0.58	MOL007207	Machiline	0.29
MOL000525	Norwogonin	0.57	MOL000497	Licochalcone a	0.29
MOL000354	Isorhamnetin	0.56	MOL004991	7-Acetoxy-2-methylisoflavone	0.25
MOL000223	Caffeic acid	0.53	MOL000057	DIBP	0.25
MOL000173	Wogonin	0.50	MOL007514	Methyl icosa-11,14-dienoate	0.24
MOL004835	Glypallichalcone	0.50	COM48	Hyperoside	0.21
MOL002560	Chrysin	0.49	MOL001490	bis [(2S)-2-ethylhexyl] benzene-1,2-dicarboxylate	0.20
MOL002295	Cinnamic acid	0.48	MOL000105	Protocatechuic acid	0.19
MOL000011	(2R,3R)-3-(4-hydroxy-3-methoxy-phenyl)-5-methoxy-2-methylol-2,3-dihydropyrano[5,6-h][1,4]benzodioxin-9-one	0.47	MOL004951	Isoliquiritin	0.17
MOL004836	Echinatin	0.47	COM24	Guanosine	0.13
MOL000874	Paeonol	0.46	MOL010921	Estrone	0.12
MOL000771	p-coumaric acid	0.46	COM47	Harmine	0.08
MOL001689	Acacetin	0.46	MOL000953	CLR	0.08
MOL001789	Isoliquiritigenin	0.45	COM25	Uridine	0.05
MOL002932	Panicolin	0.43	COM41	Caffeine	0.05
MOL002915	Salvigenin	0.41	MOL006129	6-methylgingediacetate2	0.05
MOL011319	Truflex OBP	0.40	MOL001755	24-Ethylcholest-4-en-3-one	0.04
MOL000131	EIC	0.40	COM52	Yohimbine	0.00

### Docking Simulations

To validate the effect of KFCG on stroke and further validate whether the three components, ferulic acid, vanillic acid, and zingerone, could combine with targets effectively, we conducted molecular docking by 56 components with 3D conformer and 136 protein structures responding to 49 genes in comprehensive pathways and obtained 66,967 binding relationships in the docking results (Supplementary Table S11).

**FIGURE 10:**
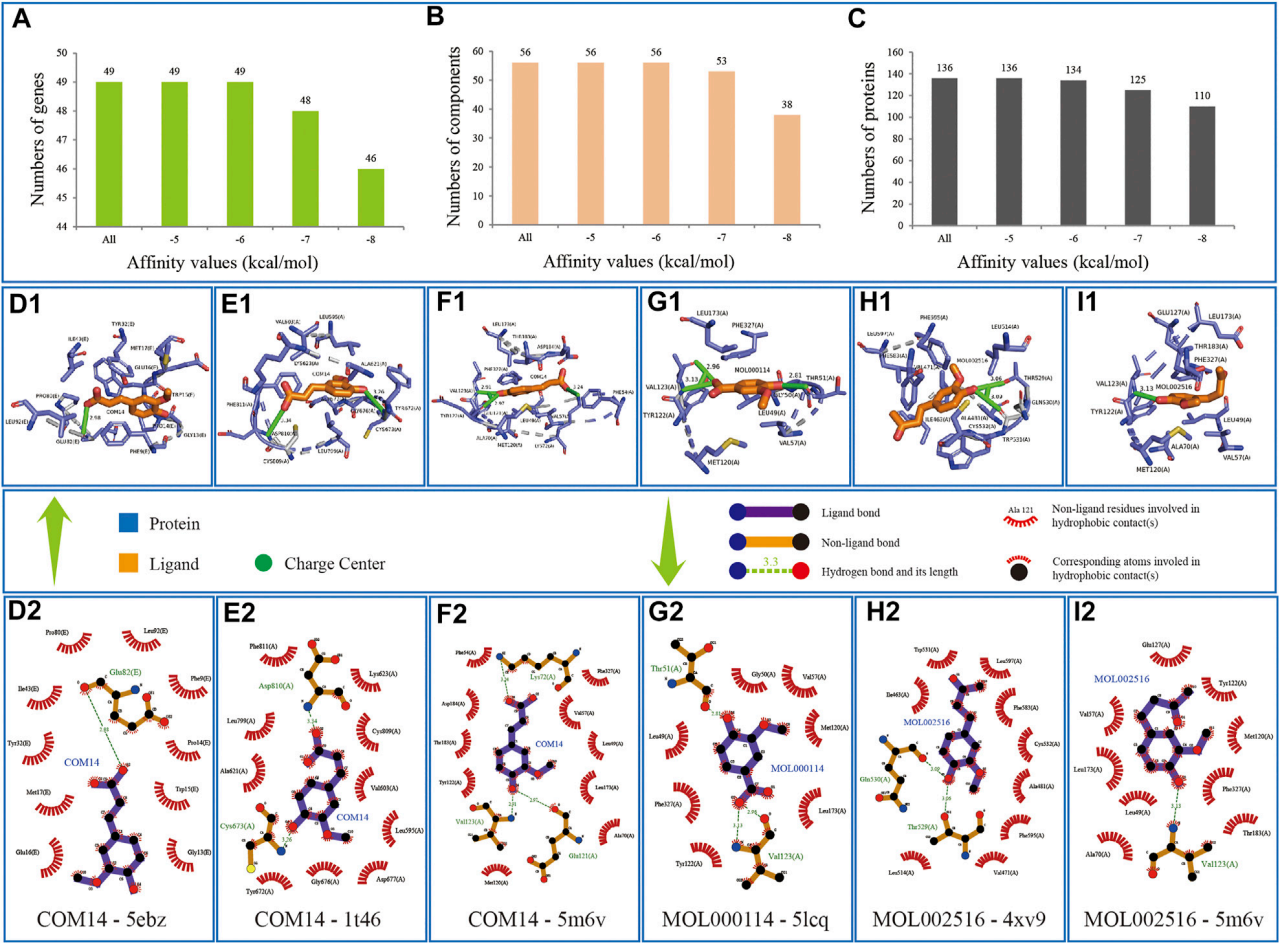
Information of docking verifications. Component is colored by elements in yellow; the combinated location between component and protein is colored by elements in green and blue. **(A–C)** represent the numbers of genes, components and proteins in different intervals of affinity values, respectively; **(D**_**1**_ **–I**_**2**_ **)** represent the bindings of COM14-5ebz, COM14-1t46, COM14-5m6v, MOL000114-5lcq, MOL002516-4xv9 and MOL002516-5m6v, respectively.

According to literatures, the lower value of affinity represents the more stable binding between protein and ligand and represent the better binding energy in protein–ligand interactions (Fu et al., 2018; Elhenawy et al., 2019). The information of binding relationships divided with binding affinity value has shown that all the 56 components of KFCG could bind the 49 genes of comprehensive pathways, whose affinities were equal to or lower than −6 kcal/mol, indicated that the 136 proteins searched from PDB could well represent the effectiveness of 49 genes in the comprehensive pathways, and confirmed that the components of KFCG could well target the proteins involved in comprehensive pathways (Figures 10A–C).

Among the 66,967 binding relationships, the three components, ferulic acid, vanillic acid, and zingerone, which were selected from the component potential effect score calculation model, showed good combinations with proteins.

Component COM14 can bind 37 genes and 68 proteins with the average binding affinity of −6.44 kcal/mol. It can bind best with protein structure 5ebz responding to gene CHUK with binding affinity of −7.6 kcal/mol, and be equal to COM14-1t46(KIT, −7.6 kcal/mol) and COM14-5m6v (PRKACA, −7.6 kcal/mol) (Figures 10D_1_–F_2_). Component MOL000114 can bind 25 genes and 37 proteins with the average binding affinity of −6.22 kcal/mol, and has a best binding relationship of MOL000114-5lcq (PRKACA, −7.2 kcal/mol) (Figures 10G_1_, G_2_). Component MOL002516 can bind 38 genes and 66 proteins with the average binding affinity of −6.40 kcal/mol, and has two best binding relationships of MOL002516-4xv9 (BRAF, −7.7 kcal/mol) and MOL002516-5m6v (PRKACA, −7.7 kcal/mol) (Figures 10H_1_–I_2_).

In addition, we found all the 56 KFCGs could have good binding ability with proteins. For example, MOL001755 can bind best with protein structure 5m6v responding to gene PRKACA with binding affinity of −11.40 kcal/mol (Supplementary Figures S2A_1_,A_2_), followed by bindings relationships of MOL010921-5m6v (PRKACA, −11.20 kcal/mol), COM52-3m2w (MAPKAPK2, −10.90 kcal/mol), and MOL000953-5m6v (PRKACA, −10.90 kcal/mol) (Supplementary Figures S2B_1_–S2D_2_). In depth, we analyzed the binding relationships whose binding affinity value is equal to or lower than −6 kcal/mol, obtained 32,928 bindings including 49 genes, 56 components, and 134 proteins, and focused on two questions including 1) which component can bind the most number of genes, 2) which genes can bind the most number of components. The results were as follows: 1) MOL000354, MOL000953, and MOL004951 can either bind 49 genes, whose average affinities are −7.30 kcal/mol, −7.33 kcal/mol, and −7.44 kcal/mol, respectively, whose optimal docking bindings are MOL000354-3zs5 (MAPK14, −10.40 kcal/mol), MOL000953-5m6v (PRKACA, −10.90 kcal/mol), MOL004951-5m6v (PRKACA, −10.70 kcal/mol), and MOL004951-5m6y (PRKACA, −10.70 kcal/mol), respectively (Supplementary Figures S2E_1_–S2H_2_). 2) Genes MAPK14 and NOS3 can both bind 56 components, whose average affinities are −7.39 kcal/mol and −7.59 kcal/mol, respectively, whose optimal docking bindings are MOL000006-3zs5 (MAPK14, −10.50 kcal/mol), MOL001689-3zs5 (MAPK14, −10.50 kcal/mol), MOL005842-3zs5 (MAPK14, −10.50 kcal/mol), and MOL010921-6pp1 (NOS3, −10.40 kcal/mol), respectively (Supplementary Figures S2I_1_–S2L_2_). The above results indicated that KFCG could effectively bind with the proteins involved in the comprehensive pathway, validating that KFCG play key roles of XXMD in the treatment of stroke.

### Experimental Validation *in Vitro*

**FIGURE 11:**
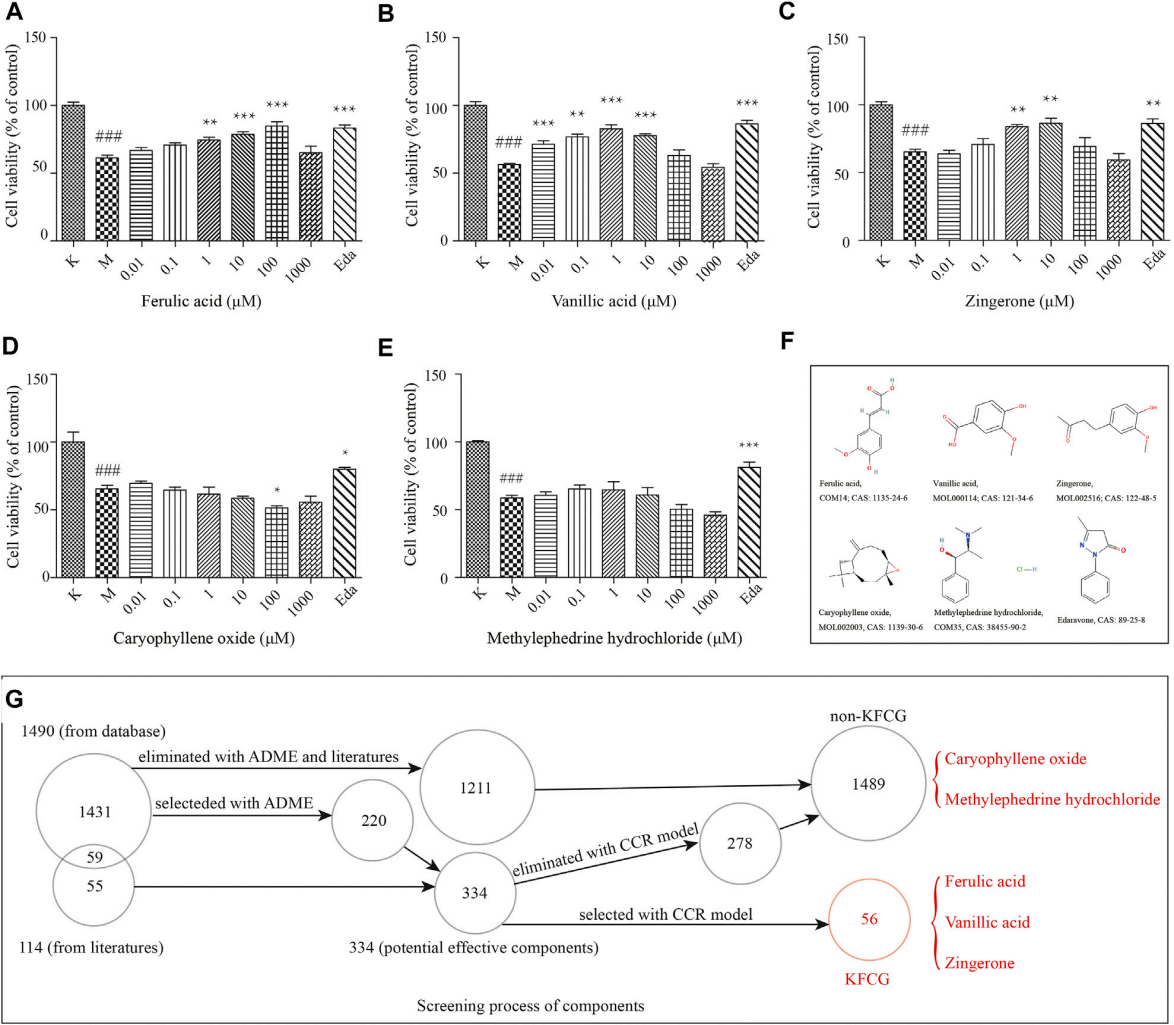
The comparation of effective components and non-KFCG components on cell viabilities induced PC12 cells. **(A–E)** The treatments with ferulic acid, vanillic acid, zingerone, caryophyllene oxide, and methylephedrine hydrochloride, respectively. **(F)** The structures of ferulic acid, vanillic acid, zingerone, caryophyllene oxide, methylephedrine hydrochloride, and edaravone. K represents the control group without the treatments of components and OGD; M represents the model group with OGD treatment and without components treatments; Eda represents edaravone (20 μM). ### represents the comparation of model group to control group (*p* = 0.001); *, **, and *** represent the comparations of components groups to model group in the levels of *p* < 0.05, *p* < 0.01, and *p* < 0.001. **(G)** The screening process of components. Five components marked in red were selected for the *in vitro* experiments.

To test the predictive power of our proposed model, we defined the 1,489 components that were eliminated by our models as the non-KFCG (Supplementary Table S9, Figure 11G). Three components of the KFCG (ferulic acid, zingerone, and vanillic acid), two components of the non-KFCG (caryophyllene oxide and methylephedrine hydrochloride), and a positive drug (edaravone) were performed in the experiments with PC12 cells (Figures 11F,G). The results showed that ferulic acid, zingerone, and vanillic acid could be protective for PC12 cells after OGD. In particular, compared with model groups, ferulic acid at 1–100 μM, vanillic acid at 0.001–10 μM, and zingerone at 1–10 μM could improve PC12 cell survival by 13.13, 17.17, 23.51, 15.02, 20.48, 26.46, 21.45, 18.60, and 21.14%, respectively, at a level comparable to edaravone treatment (Figures 11A–C). Relatively, the two components of the non-KFCG which were eliminated with our model, caryophyllene oxide and methylephedrine hydrochloride, could not protect PC12 cells (Figures 11D,E), while edaravone could significantly protect PC12 cells. At the same time, the results in the experiments with HT22 cells also showed that ferulic acid, zingerone, and vanillic acid could improve the survival of HT22 cells after OGD (Supplementary Figure S3, Supplementary Methods and Materials).

These results in the *in vitro* experiments proved that our proposed model could be used in selecting the KFCG of XXMD in treating stroke, effectively and accurately.

## Discussion

The main purpose of formula optimization is to reduce the non-pharmacological factors and improve the curative effect of the formula. According to the theory of TCM, different medicinal components are composed as the formula. But whether all of these components are necessary in treating disease still needs further analysis and verifications. During the optimization of formula, some medicine components with certain drug effects can be screened out, and thus the material composition and the effect of formula will become more simplified and clarified.

XXMD has a long history in China. Both clinical data and animal experimental evidence have confirmed that XXMD can effectively promote the treatment of stroke (Ye et al., 1999; An et al., 2019; Liu et al., 2021). However, based on the current research status of TCM in China, the qualities of RCT study of many Chinese herbal formulas, including XXMD, still need to be improved. For providing more reliable suggestions at clinical treatment, the complex mechanism of these formulas in treating diseases still needs to be studied and explained. XXMD is a traditional adjuvant treatment of stroke and is with the traditional dose of 600 ml. But it is usually used based on the condition of the state of stroke and the personal physical quality of patients. For example, children are usually advised to take a half of the dose, and the patient whose condition improves very well is usually advised to appropriately take less dose of XXMD. However, we did not find the detailed reports which studied that the heavier patients should get larger doses, and neither found the detail reports which showed the relationship between the patients’ condition and the doses of XXMD the patients should get, while the clinical applications are sometimes mentioned in some literatures (Zhang et al., 2021). Even though there are not enough randomized controlled trials to clearly prove and judge the user population in most of the current studies, XXMD has been listed in the first batch of classic prescriptions in China (Cai et al., 2007; Fu et al., 2013; Luo X. et al., 2019; Jia et al., 2019) and is still to be recommended with its effectiveness and safety, which is consistent with the views of some researchers (Fu et al., 2013; Fu and Cai, 2016; Chen Q. R. et al., 2017; Jiang and Li, 2020; Huang et al., 2021).

To better optimize the classical formula XXMD for the treatment of stroke, network pharmacology and mathematical methods were employed to investigate relatively optimal KFCG. Network pharmacology has the characteristics of system and integrity, which is in line with the philosophical view of “Chinese medicine is holistic” in Chinese medicine research. At present, network pharmacology has been widely used in the treatment of complex diseases by TCM. It emphasizes the concept of multi-target regulation of signaling pathways and helps to improve the therapeutic effect of drugs and reduce toxic and side effects. For example, network pharmacology can contribute to study the potential molecular mechanisms of TCM prescriptions in the treatment of complex diseases, such as “treating different diseases with the same treatment, treating the same diseases with different treatment.” However, there are a few reports on the optimization of TCM prescriptions based on network pharmacology, especially in the study of the classical formula XXMD.

In our study, we propose an integrative strategy to optimize XXMD, obtain the key components of XXMD, and analyze the potential mechanism of these components with comprehensive pathways and docking simulations. Our approach has two advantages:

1) In this study, we proposed a new method for calculating the importance of nodes, which takes the connectivity and radiation influence of node in the network into account. Based on this method (Figure 6), we determined the FRS and effective proteins of XXMD. The effective proteins can be enriched into 99.10% KEGG pathways from UETs of XXMD, which is higher than those by degree, ClosenessCentralty and ClusteringCoefficient with 0.90, 1.80, and 15.32%, respectively (Figure 6A). Meanwhile, compared with the methods of degree, ClosenessCentralty and ClusteringCoefficient, effective proteins in FRS determined by our method can be enriched into 97.30% GO-terms of genes from the UETs in XXMD, which is higher with 3.02, 3.14, and 20.9%, respectively (Figure 6B). These results showed that the functional response selection method is accurate and reliable.
2) In recent years, network pharmacology provides a powerful tool for exploring the compatibility and mechanism of the TCM formula but with some limitations. For example, there are still lack researches and recommendations on the optimization of XXMD pharmaceutical components even though wet-lab experiments, which are often used in validating the efficacy and potential mechanism of XXMD in the treatment of stroke. In this study, we obtained better performance in the XXMD components optimization.

Based on the new model proposed and used in this study, we deeply optimized the formula and obtained the KFCG of XXMD, which can represent the effect of XXMD in treating stroke. We used comprehensive assessments to confirm that KFCG could represent the molecular effect of XXMD. Based on the traditional functional annotations of targets, pathogenetic genes, and KFCG targets, we calculated the functional coverage obtained in our reverse optimization model. As a result, it was found that KFCG could respond well to the combination effects of different chemical components extracted from various herbs in XXMD. The advantages of this study are that FRS and the component reverse search strategy are applied to find the KFCG of XXMD, providing a methodological reference to the study and development of TCM.

Based on the FRS, we optimized the contribution score by using the CCR model, and finally obtained KFCG with 56 components. Enriched pathways of KFCG targets can cover 80.43% of the enriched pathways of pathogenetic genes, respectively (Figure 8), meaning that the targets of these KFCG are closely related to pathogenetic genes. It validated the reliability of our FRS and CCR model once again. Furthermore, the targets of the top seven components in KFCG (vanillic acid, ferulic acid, L-tryptophan, Dihydrocapsaicin, zingerone, rhamnetin, Norwogonin) of XXMD could cover 50.00% of effective proteins (Figure 7), providing a strong reference for other formula optimization. Some of these components had been proven to be beneficial for the recovery of stroke or to have indirective effect on the neuroprotection. For example, ferulic acid treatment could protect the brain against cerebral ischemic injury by preventing the ischemic injury-induced increases of caspase-3 and the ischemic injury-induced decrease in hippocalcin expression (Koh, 2013). Zingerone had been proven to increase genes expression in the Notch pathway which could promote proliferations of neural stem cells and enhance hippocampal neurogenesis (Zhang et al., 2008; Zhang et al., 2012; Davis and Rajanikant., 2020). Vanillic acid has an indirective effect on the neuroprotection (Salau et al., 2020b). In addition, molecular docking indicated that KFCG could bind the proteins involved in comprehensive pathways effectively, such as COM14, MOL000114, MOL002516, MOL001755, MOL010921, COM52, and MOL000953 (Figure 10D_1_-10I_2_, Supplementary Figure S2A1-S2D21). These components in KFCG showed a potential contribution to the treatment of stroke by KFCG (Figure 9) (Li et al., 2015; Li et al., 2016; Qian et al., 2016; Kempuraj et al., 2020). It is indicated that KFCG can represent the key function of XXMD in treating stroke and the CCR model used in our study was effective for XXMD optimization.

Currently, due to the limitations of experimental conditions, the content of components in decoction and the role of these components in organisms still need more studies. Reviewing the *in vitro* experiments in this study, we estimated that the three components, zingerone, ferulic acid, vanillic acid, could be effectively absorbed and transferred to the brain mainly based on some reports and their properties provided from the TCMSP database (https://old.tcmsp-e.com/load_intro.php?id=29). For example, zingerone had been detected in XXMD even though its concentration had not been reported (Li et al., 2006). Zingerone had been reported to rapidly cross the BBB and metabolize in rodents easily (Rashid et al., 2021). Ferulic acid had been proved to have the concentration of 58.32 μg/ml in XXMD (Xiao, 2008), and could be transported to plasma and brain of rats from XXMD (Wang et al., 2016). Furthermore, the ability of ferulic acid at crossing the blood–brain barrier (BBB) was predicted *in silico* using the SwissADME online serve (Salau et al., 2020a). Even though there is a lack of the concentrations reports of vanillic acid in XXMD, some animal experiments have confirmed that vanillic acid can be absorbed after oral administration of some formula, such as Chaigui granules (Gao et al., 2020a), Jiao-Tai-Wan (Li Z. et al., 2020), dispensing granules, and standard decoction of *Cinnamomum cassia* twigs (Tao et al., 2019). After the oral administration, vanillic acid can be detected in plasma (Tao et al., 2019; Li Z. et al., 2020), ileum and brain (Gao et al., 2020a), and has been proved to play the neuroprotective roles in the rat models by decreasing the levels of malondialdehyde (Ahmadi et al., 2021a), IL-6 and TNF-α (Khoshnam et al., 2018), by increasing the levels of IL-10 (Khoshnam et al., 2018) and total thiol group (TTG) (Ahmadi et al., 2021a), and by suppressing oxidative stress (Ahmadi et al., 2021b). Furthermore, according to the information provided in TCMSP, the compounds with BBB < −0.3 were considered nonpenetrating (BBB−), from −0.3 to +0.3 moderate penetrating (BBB±), and >0.3 strong penetrating (BBB+). Thus, ferulic acid and zingerone were considered to have strong penetrating with the values of BBB = 0.56 and BBB = 0.48, respectively. Vanillic acid was considered to have the moderate penetrating with the value of BBB = 0.09.

At the same time, we estimate that these three components, ferulic acid, vanillic acid, and zingerone, have the concentrations of 227.76 nM, 0.34, and 2.07 μM in the brain, respectively, after eating a dose of XXMD herbs. The calculation is done based on some backgrounds: 1) We presume that components can be 100% extracted and 100% absorbed, and presume that the weight of a human is 70 kg. 2) CX, RS, and SJ are 50, 50, and 250 g in a dose of XXMD herbs, respectively. The proportions of ferulic acid in CX and RS are 0.19 mg/g and 32.70 mg/g, respectively (Table 1). The proportion of vanillic acid in CX is 0.08 mg/g (Table 1). The proportion of zingerone in SJ is 27.30 mg/g (Table 1). 3) The molecular weights of ferulic acid, vanillic acid, and zingerone are 194.18 g/mol, 168.15 g/mol, and 194.23 g/mol, respectively. 4) Ferulic acid, vanillic acid, and zingerone can be soluble into water with the solubility value of −1.42, −1.32, and −3.1, respectively, according to the SwissADME database (http://www.swissadme.ch/), whereas the solubility value of −2∼0 represents very easily soluble and −4∼−2 represents soluble. These three components have been proved to across BBB and into brain (Wang et al., 2016; Gao et al., 2020a; Rashid et al., 2021). 5) Reports showed that the rat (200 g) could get 45.00 ng/g concentration in the brain after the feeding of 10.90 mg/kg zingerone (Li L.-L. et al., 2019), the rat (200 g) could get 120.01 ng/g concentration in the brain after the feeding of 63.75 mg/kg ferulic acid (Mi et al., 2020), and the mice (25 g) could get 30.00 μg/g concentration in the brain after the injection of 30.00 mg/kg vanillic acid (Amin et al., 2017).

During the comparation between these estimations and the results in the *in vitro* experiments, we can know that the concentration 0.34 μM of vanillic acid is located in the concentration range which has the significant promotion for the survival of PC12 cells (Figure 11B), whereas vanillic acid could significantly promote the survival of PC12 cells in the concentrations of 0.01, 0.1, 1, and 10 μM. The concentration 2.07 μM of zingerone is located in the concentration range which has the significant promotion for the survival of PC12 cells (Figure 11C), whereas zingerone were proved to be significantly promoting the survival of PC12 cells in the concentrations of 1 and 10 μM. However, the concentration 227.76 nM of ferulic acid had not been proved to be that whether it could significantly promote the survival of PC12 cells (Figure 11A), while ferulic acid could significantly promote the survival of PC12 cells in the concentrations of 1 μM, 10, and 100 μM.

For why the concentration 227.76 nM of ferulic acid had not been proved to be that whether they could significantly promote the survival of PC12 cells (Figure 11A), we estimate that there are some reasons: 1) The concentration 227.76 nM of ferulic acid is located in the range of 0.1–1 μM, while 1 μM was proved to promote the survival of PC12 cells and 0.1 μM was not proved to promote the survival of PC12 cells (Figure 11A). Thus, we could not be sure whether the concentration 227.76 nM of ferulic acid could promote the survival of PC12 cells. 2) The estimation of the concentration 227.76 nM is calculated based on the values that were provided in the experiments of rats. The accuracy of these values is affected by the experiments in these cited articles. As results, these values can only be used as a reference, and cannot be used as a basis for the complete and accurate calculation or prediction of the results of ferulic acid in PC12 cell experiments. 3) There may be differences between the efficacy of a compound and that of a single component. For getting more accurate concentrations into the brain, we need to further determine the components with rat experiments and with the methods of metabolomics, proteomics, transcriptomics, etc.

In our study, there are two original purposes: 1) the main purpose of our study is to put forward new methods and build new models for the compound optimization of XXMD in treating stroke. We hope to provide reference for the study of key functional components of XXMD in treating stroke. 2) After screening the key functional components, the *in vitro* experiments were taken to verify whether some of these key functional components could promote cells’ survival. In the results of our study, these two purposes had been achieved. Here, the estimation of the effective concentration of these three components, ferulic acid, vanillic acid, and zingerone, in the brain can be used as an expanded thinking. We did not mainly compare whether the concentrations of the three components (ferulic acid, vanillic acid, and zingerone) into the brain which were estimated based on literatures could also significantly promote the survival of PC12 cells because of that the concentrations into the brain were estimated based on some literatures and their accuracy needs further studies. Results in PC12 cells can be used as a reference for the further study of the key functional components of XXMD. Relatively, if it is necessary to further study whether the key functional components have clinical effect in treating stoke, these components need to provide with further and in-depth verifications and experiments, which is one of the aspects of our efforts in the future.

During the *in vitro* experiments, the experiment with PC12 cells showed a better dose–response (Figure 11). For the reason why the large dose of components is ineffective when compared with disease model group M (ferulic acid at 1,000 μM, vanillic acid at 100 and 1000 μM, zingerone at 100 and 1,000 μM), we speculate that this may be a combined effect of OGD and compound-toxicity to cells. The results of the *in vitro* experiments with PC12 cells and HT22 cells (Figure 11, Supplementary Figure S3, Supplementary Methods and Materials) showed the good protective effect of these three components on cells after OGD, indicating that our proposed model could be used in selecting the KFCG of XXMD in treating stroke, effectively and accurately. Based on our proposed model, we can also find some components that have the potential effect in treating stoke. For example, there is still a lack in the study of vanillic acid in treating stroke (Khoshnam et al., 2018). In this study, three components (ferulic acid, vanillic acid, and zingerone) and their potential effective concentrations were preliminarily studied. In the future, finding more effective components and the more accurate concentrations that can be used in the human body is a direction of our efforts.

On the whole, we proposed a reverse optimization model based on the association of pathogenetic genes and component targets to improve the accuracy on decoding KFCG of XXMD, providing reference for the optimization and mechanism analysis of the formula in TCM.

## Data Availability

The original contributions presented in the study are included in the article/Supplementary Material, further inquiries can be directed to the corresponding authors.

## Supplementary Material

Supplementary Figure S1 The structure of 56 components in KFCG.

Supplementary Figure S2 Twelve bindings relationships.

Supplementary Figure S3 Experimental vialidation in HT22 cells. Validation of effective components on HT22 cells. **(A–C)** the treatments with ferulic acid, vanillic acid and zingerone, respectively. K represents the control group without the treatments of components and OGD; M represents the model group with OGD treatment and without components treatments; ### represents the comparation of model group to control group (*p* = 0.001); *** represents the comparations of components groups to model group in the levels of *p* < 0.001.

Supplementary Methods and Materials Experimental validation in HT22 cells.

Supplementary Table S1 Pathogenetic genes. “Genes” represent the name of genes; “Numbers of Literatures” represent the numbers of literatures which have reported the effect of gene in the stroke; “Description” represents the traditional name of gene; “Genes Link in GeneCard Database” is the link of the detail description of gene in the GeneCard.

Supplementary Table S2 Components of 12 herbs.

Supplementary Table S3 220 components selected by ADME conditions from TCMSP.

Supplementary Table S4 334 Potential active components.

Supplementary Table S5 Nine components of the shared components of herbs in XXMD.

Supplementary Table S6 Specific components of herbs in XXMD.

Supplementary Table S7 334 potential active components and 1,329 targets.

Supplementary Table S8 Disease-targets network.

Supplementary Table S9 The 1489 components of non-KFCG.

Supplementary Table S10 Targets of KFCG.

Supplementary Table S11 Information of docking simulations.

## Glossary

ACE: Angiotensin converting enzyme
AD: Alzheimer’s disease
ADME: Absorption, distribution, metabolism and excretion
AR: Androgen receptor system
AS: Atherosclerosis
BDNF: Brain-derived neurotrophic factor
BS: *Paeonia lactiflora* Pall
BYHWT: Buyang Huanwu Tang
Caco-2: Caco-2 permeability
CADASIL: Cerebral autosomal dominant arteriopathy with subcortical infarcts and leukoencephalopathy
CI: Contribution index
CREB: cAMP response element binding protein
C-T: Component-target
C-T-P: Components-targets-pathways
CX: *Ligusticum striatum* DC
DL: Drug-likeness
EMF: Ermiao fang
EVs: Extracellular vesicles
FF: *Saposhnikovia divaricata* (Turcz.) Schischk
FJ: *Stephania tetrandra* S.Moore
FZ: *Aconitum wilsonii* Stapfex Veitch
GC: *Glycyrrhiza uralensis* Fisch
GO: Gene Ontology
GSK-3: Glycogen synthase kinase-3
HLD: Huanglian Decoction
HLJDD: Huang-Lian-Jie-Du Decoction
HQ: *Scutellaria baicalensis* Georgi
ICH: Intracerebral hemorrhage
IL-6: Interleukin-6
IS: Ischemic stroke
NIHSS: National Institute of Health stroke scale
KEGG: Kyoto Encyclopedia of Genes and Genomes
KFCG: Key functional components group
KXR: *Amygdalus communis* L
Map2: Microtubule-associated protein 2
MDA: Malondialdehyde
MEK: Mitogen-active protein/extracellular signal-regulated kinase
MH: *Ephedra alata* Decne
MTHFR: Methylene tetrahydrofolate reductase
MW: Molecular weight
NO: Nitric oxide
OB: Oral bioavailability
OGD: Oxygen and glucose deprivation
PDE4D: Phosphodiesterase 4D
PES: Potential effect score
PPI: Protein-protein interaction
QKL: Qing-Kai-Ling
RAS: Renin-angiotensin system
RG: *Cinnamomum cassia* (L.) J. Presl
RS: *Ginseng quinquefolium* (L.) Alph.Wood
rt-PA: Recombinant tissue-type plasminogen activator
SEA: Similarity Ensemble Approach
SJ: *Zingiber officinale* Roscoe
T2DM: Type 2 diabetes
TCM Database @Taiwan: traditional Chinese medicine database@Taiwan
TCM: Traditional Chinese medicine
TCMSP: traditional Chinese medicine systems pharmacology database and analysis platform
TFs: Transcription factors
TLR4/NF-κB: TLR4 of toll-like receptor 4/nuclear factor kappa B
UETs: Unoptimized effective targets
VEGF: Vascular endothelial growth factor
XXMD: Xiao-Xu-Ming decoction
YD: Yinlai Decoction
