# Corrigendum: Detecting key functional components group and speculating the potential mechanism of Xiao-Xu-Ming decoction in treating stroke

**DOI:** 10.3389/fcell.2022.1107236

**Published:** 2023-01-20

**Authors:** Yu-peng Chen, Ke-xin Wang, Jie-qi Cai, Yi Li, Hai-lang Yu, Qi Wu, Wei Meng, Han-duo Wang, Chuan-hui Yin, Jie Wu, Mian-bo Huang, Rong Li, Dao-gang Guan

**Affiliations:** ^1^ Department of Biochemistry and Molecular Biology, School of Basic Medical Science, Southern Medical University, Guangzhou, China; ^2^ Guangdong Provincial Key Laboratory of Single Cell Technology and Application, Southern Medical University, Guangzhou, China; ^3^ Guangdong Provincial Key Laboratory on Brain Function Repair and Regeneration, Department of Neurosurgery, National Key Clinical Specialty/Engineering Technology Research Center of Education Ministry of China, Neurosurgery Institute, Zhujiang Hospital, Southern Medical University, Guangzhou, China; ^4^ Department of Radiology, Nanfang Hospital, Southern Medical University, Guangzhou, China; ^5^ Department of Burns, Nanfang Hospital, Southern Medical University, Guangzhou, China; ^6^ Department of Histology and Embryology, Guangdong Provincial Key Laboratory of Construction and Detection in Tissue Engineering, School of Basic Medical Sciences, Southern Medical University, Guangzhou, China; ^7^ Department of Cardiovascular Disease, First Affiliated Hospital of Guangzhou University of Chinese Medicine, Guangzhou, China

**Keywords:** XXMD, KFCG, network pharmacology, functional response space, effective proteins, PC12 cells, HT22 cells

In the published article, there was an error in the author list, and author Jie-qi Cai was erroneously not listed as a co-first author. The corrected author list appears below.

“Yu-peng Chen^†^, Ke-xin Wang^†^, Jie-qi Cai^†^, Yi Li, Hai-lang Yu, Qi Wu, Wei Meng, Han-duo Wang, Chuan-hui Yin, Jie Wu, Mian-bo Huang*, Rong Li*, Dao-gang Guan*.”

In the published article, there was an error. A correction has been made to **AUTHOR CONTRIBUTIONS**. This sentence previously stated:

“D-gG, RL, and M-bH provided the concept and designed the study. Y-pC conducted the analyses and wrote the manuscript. K-xW, J-qC, YL, H-lY, QW, WM, H-dW, C-hY, and JW participated in data analysis. D-gG, RL, and M-bH contributed to revising and proof-reading the manuscript. All authors read and approved the final manuscript.”

The corrected sentence appears below:

“D-gG, RL, and M-bH provided the concept and designed the study. Y-pC conducted the analyses and wrote the manuscript. K-xW, J-qC participated in data analysis, figures designs and revision, YL, H-lY, QW, WM, H-dW, C-hY, and JW participated in data analysis. D-gG, RL, and M-bH contributed to revising and proof-reading the manuscript. All authors read and approved the final manuscript.”

The authors apologize for this error and state that this does not change the scientific conclusions of the article in any way. The original article has been updated.

