# Supplementary figures and images for: Detecting Key Functional Components Group and Speculating the Potential Mechanism of Xiao-Xu-Ming Decoction in Treating Stroke
Source: Front Cell Dev Biol. 2022 May 12;10:753425. doi: 10.3389/fcell.2022.753425 (PMC9136080; doi:10.3389/fcell.2022.753425)

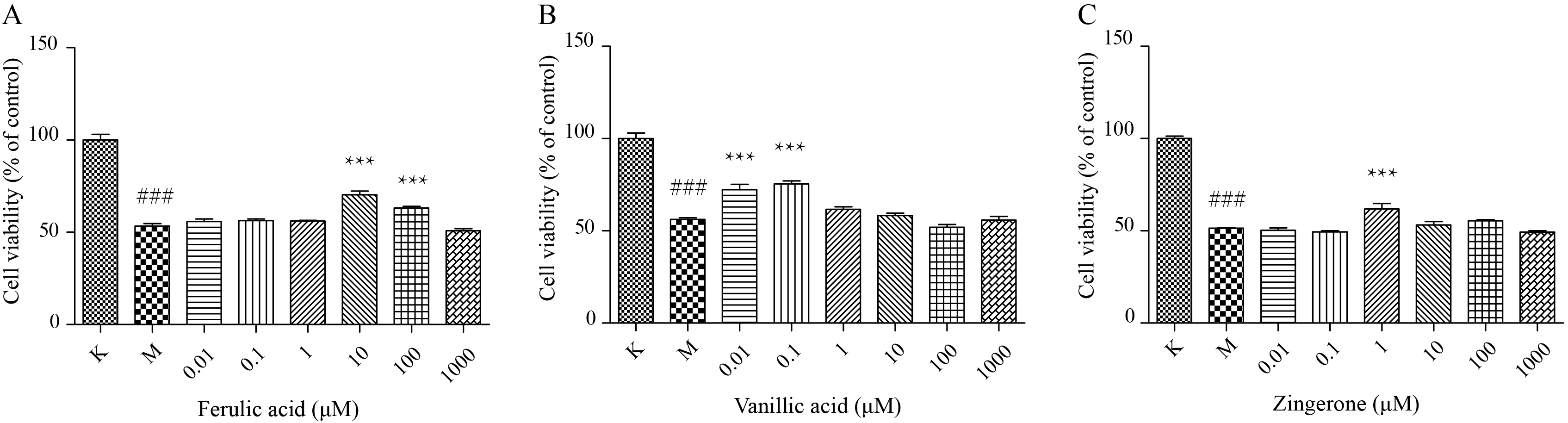

Supplement: Supplementary file 2 [file Image3.jpeg]

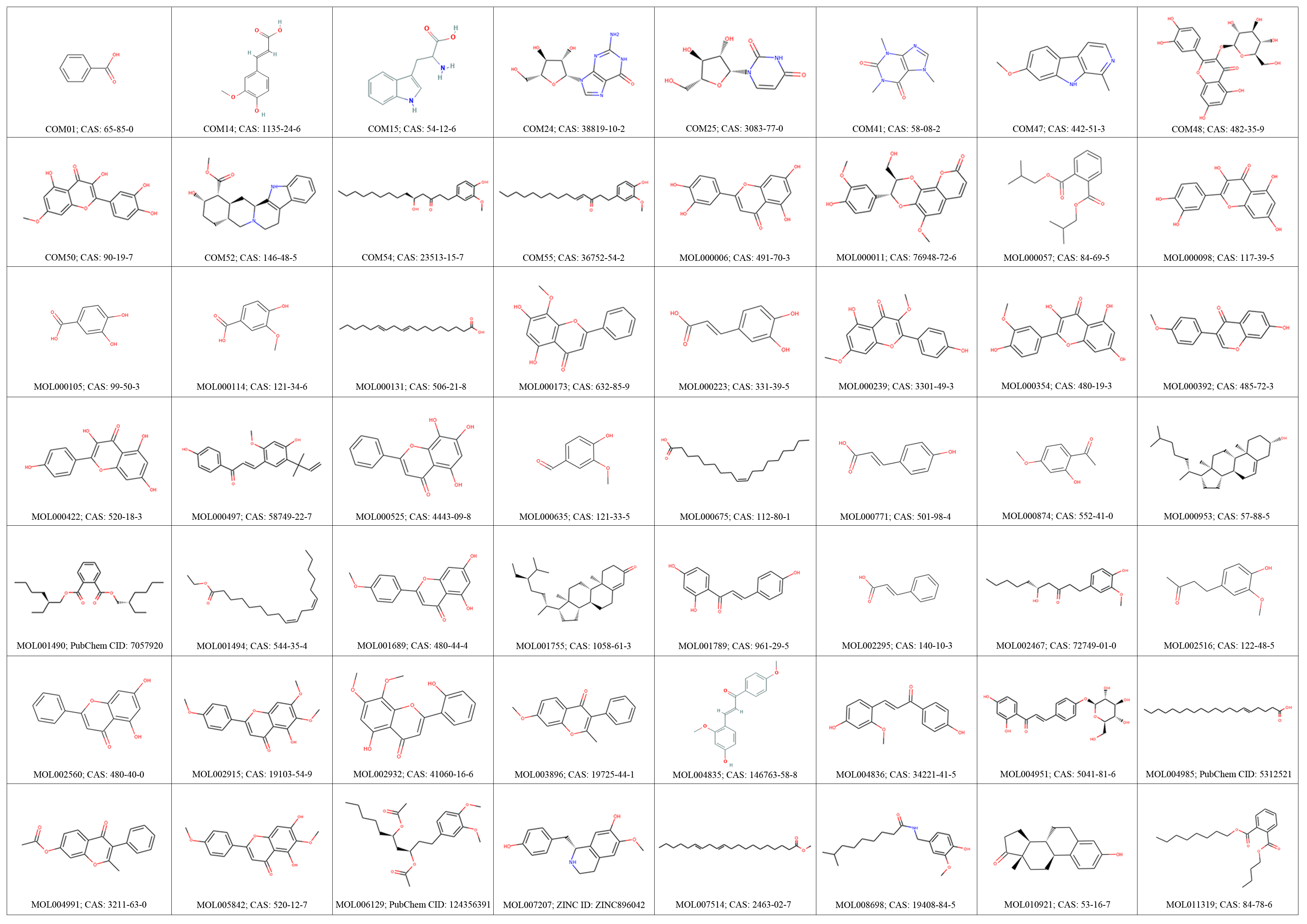

Supplement: Supplementary file 6 [file Image1.jpeg]

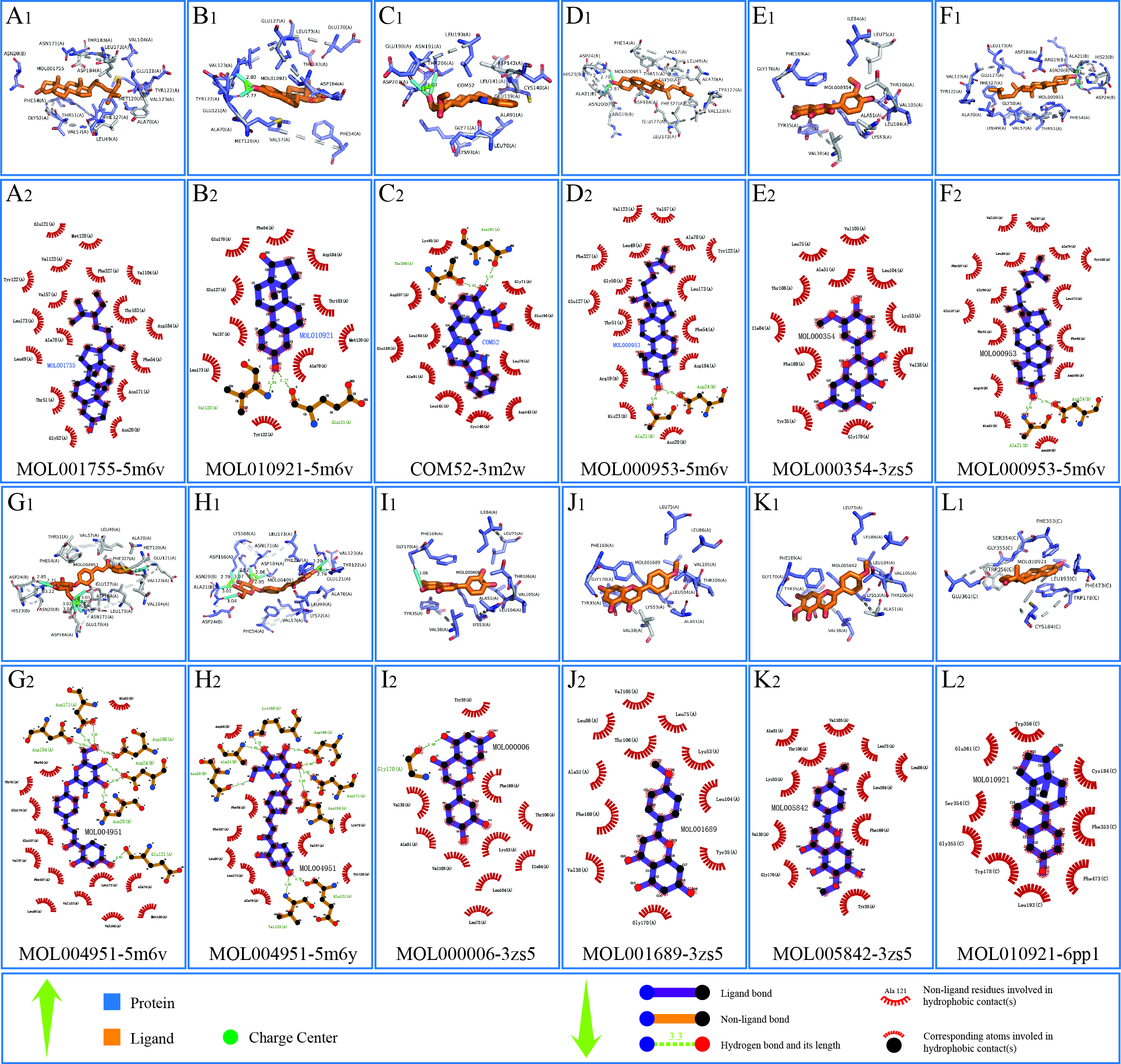

Supplement: Supplementary file 7 [file Image2.jpeg]
