# Supplementary material for: Detecting Key Functional Components Group and Speculating the Potential Mechanism of Xiao-Xu-Ming Decoction in Treating Stroke
Source: Front Cell Dev Biol. 2022 May 12;10:753425. doi: 10.3389/fcell.2022.753425 (PMC9136080; doi:10.3389/fcell.2022.753425)
Supplement: Supplementary file 4 [file Presentation1.zip › Supplementary Methods and Materials_Experimental validation in HT22 cells.DOCX]

**Supplementary Methods and Materials**

**Experimental Validation in HT22 cells**

**MATERIALS AND METHODS**

**Materials**

Fetal bovine serum (FBS) and Dulbecco’s Modified Eagle’s Medium (DMEM) were purchased from ThermoFisher Biochemical Products (Beijing) Co., Ltd. Hypoxic bags were purchased from Mitsubishi Gas Chemical Company, Inc (Japanese). Ferulic acid (≥ 98% purity by HPLC), zingerone (≥ 98% purity by HPLC) and vanillic acid (≥ 97% purity by HPLC) were purchased from Jiangsu Yongjian Pharmaceutical Technology Co., Ltd (Jiangsu, China). Cell Counting Kit-8 (CCK-8) was purchased from Dojindo Laboratories (Japanese).

**Cell Culture**

HT22 neuronal cell line of mouse hippocampus was obtained from CHI SCIENTIFIC (Shanghai, China) and cultured in DMEM with FBS, penicillin 100 U/mL, streptomycin 100 μg/mL, respectively, at 37 ℃ in a fully humidified 5% CO_2_ atmosphere.

**Oxygen and Glucose Deprivation Protocol**

Oxygen and glucose deprivation (OGD) is a well-established *in vitro* model in studying the pathology and pharmacology of ischemic damage ([Gu et al., 2013](#_ENREF_1); [Guo et al., 2013](#_ENREF_2); [Morán et al., 2017](#_ENREF_6); [Tian et al., 2020](#_ENREF_11)). Considering the actual clinical situations, patients usually take medications of XXMD after the occurrence of stroke ([Pan et al., 2017](#_ENREF_8); [Zhong et al., 2020](#_ENREF_12)), we study the effect of components in treating stroke based on HT22 cells in the case that cells were protected with components after OGD. During the OGD, cells were incubated in culture medium without FBS in a hypoxic bag at 0.1% O_2_, 5% CO_2_, and 37 ℃ for 18h ([Gu et al., 2013](#_ENREF_1); [Morán et al., 2017](#_ENREF_6)).

**Validation of Effective Components in HT22 Cells**

CCK-8 assay was utilized to measure cell viability. Cells were seeded in 96-well plates (2 × 10^4^ per/well). After 24 h incubation, HT22 cells were treated without any components for 18 h in OGD. A control group without the treatments of OGD and components was taken at the same time. After the OGD period, cells were treated with 0.01 μM, 0.1μM, 1 μM, 10 μM, 100 μM and 1000 μM ferulic acid ([Hassanzadeh et al., 2018](#_ENREF_3); [Moghadam et al., 2018](#_ENREF_5); [Nakayama et al., 2020](#_ENREF_7)), zingerone ([Ho et al., 2013](#_ENREF_4); [Ruangsuriya et al., 2017](#_ENREF_9)) and vanillic acid ([Shen et al., 2018](#_ENREF_10)), and were incubated with complete culture medium under normoxic condition for 18 h. Furthermore, a model group with OGD treatment and without components treatments was taken at the same time. Then, cells were changed to be cultured in fresh complete culture medium with ten μl of CCK-8 for a further 4 h. The absorbance was measured at 450 nm with a microplate reader. The experiments were repeated with 5 replicates.

**Statistical Analysis**

All data were expressed as mean ± SEM. The differences between model group and control group were analyzed by student t test. The differences between components treatments and model group were analyzed by one-way ANOVA for multiple comparisons. Results were considered as statistically significant if the p-value was < 0.05.

**RESULTS**

**Experimental Validation *in Vitro***

To test the predictive power of our proposed model, three components in KFCG, ferulic acid, zingerone and vanillic acid were taken to the *in vitro* experiments with HT22 cells. Result showed that, compared to disease model control, ferulic acid could increase the viabilities of HT22 in concentrations of 10μM and 100μM by 17.08% and 9.88% (Supplementary Figure S3A); vanillic acid could increase the viabilities of HT22 in concentrations of 10nM and 100nM by 16.13% and 19.23% (Supplementary Figure S3B); zingerone could increase the viabilities of HT22 in concentrations of 1μM by 10.39% (Supplementary Figure S3C).


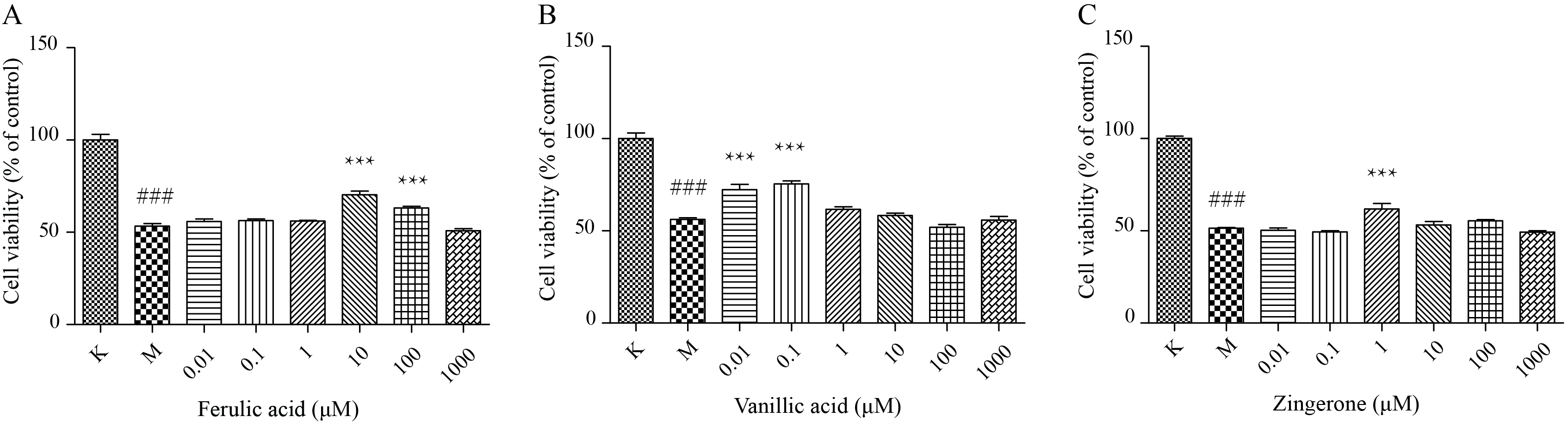


<Supplementary Figure S3. Insert here>

**Supplementary Figure S3.** Validation of effective components on HT22 cells. (A-C) the treatments with ferulic acid, vanillic acid and zingerone, respectively. K represents the control group without the treatments of components and OGD; M represents the model group with OGD treatment and without components treatments; ### represents the comparation of model group to control group (p=0.001); *** represents the comparations of components groups to model group in the levels of p < 0.001.

These results in the *in vitro* experiments proved that our proposed model could be used in selecting the KFCG of XXMD in treating stroke, effectively and accurately.

**References**

Gu, J.H., Ge, J.B., Li, M., Xu, H.D., Wu, F., and Qin, Z.H. (2013). Poloxamer 188 protects neurons against ischemia/reperfusion injury through preserving integrity of cell membranes and blood brain barrier. *PLoS One* 8(4)**,** e61641. doi: 10.1371/journal.pone.0061641

Guo, F., Wang, H., Li, L., Zhou, H., Wei, H., Jin, W., et al. (2013). A novel domain of amino-Nogo-A protects HT22 cells exposed to oxygen glucose deprivation by inhibiting NADPH oxidase activity. *Cell Mol Neurobiol* 33(3)**,** 443-452. doi: 10.1007/s10571-013-9911-1

Hassanzadeh, P., Arbabi, E., Atyabi, F., and Dinarvand, R. (2018). Ferulic acid-loaded nanostructured lipid carriers: A promising nanoformulation against the ischemic neural injuries. *Life Sci* 193**,** 64-76. doi: 10.1016/j.lfs.2017.11.046

Ho, S.C., Chang, K.S., and Lin, C.C. (2013). Anti-neuroinflammatory capacity of fresh ginger is attributed mainly to 10-gingerol. *Food Chem* 141(3)**,** 3183-3191. doi: 10.1016/j.foodchem.2013.06.010

Moghadam, F.H., Mesbah-Ardakani, M., and Nasr-Esfahani, M.H. (2018). Ferulic Acid exerts concentration-dependent anti-apoptotic and neuronal differentiation-inducing effects in PC12 and mouse neural stem cells. *Eur J Pharmacol* 841**,** 104-112. doi: 10.1016/j.ejphar.2018.10.003

Morán, J., Perez-Basterrechea, M., Garrido, P., Díaz, E., Alonso, A., Otero, J., et al. (2017). Effects of Estrogen and Phytoestrogen Treatment on an In Vitro Model of Recurrent Stroke on HT22 Neuronal Cell Line. *Cell Mol Neurobiol* 37(3)**,** 405-416. doi: 10.1007/s10571-016-0372-1

Nakayama, H., Nakahara, M., Matsugi, E., Soda, M., Hattori, T., Hara, K., et al. (2020). Protective Effect of Ferulic Acid against Hydrogen Peroxide Induced Apoptosis in PC12 Cells. *Molecules* 26(1). doi: 10.3390/molecules26010090

Pan, Y., Zhang, W., Zhang, W., Bai, X., Ren, S., Zheng, J., et al. (2017). Pharmacokinetic comparison of two phenolic acids after oral administration of Typhae pollen to normal rats and rats with acute cold blood stasis. *Biomed Chromatogr* 31(12). doi: 10.1002/bmc.4028

Ruangsuriya, J., Budprom, P., Viriyakhasem, N., Kongdang, P., Chokchaitaweesuk, C., Sirikaew, N., et al. (2017). Suppression of Cartilage Degradation by Zingerone Involving the p38 and JNK MAPK Signaling Pathway. *Planta Med* 83(3-04)**,** 268-276. doi: 10.1055/s-0042-113387

Shen, L., Jiang, H.H., and Ji, H.F. (2018). Is boiled food spice curcumin still biologically active? An experimental exploration. *Food Nutr Res* 62. doi: 10.29219/fnr.v62.1397

Tian, R., Wu, B., Fu, C., and Guo, K. (2020). miR-137 prevents inflammatory response, oxidative stress, neuronal injury and cognitive impairment via blockade of Src-mediated MAPK signaling pathway in ischemic stroke. *Aging (Albany NY)* 12(11)**,** 10873-10895. doi: 10.18632/aging.103301

Zhong, D.Y., Li, H.Y., Li, L., Ma, R.M., Jiang, C.T., Li, D.X., et al. (2020). Effect of Tongqiao Huoxue Decoction Combined with Western Medicine on Ischemic Stroke: A Systematic Review. *Evid Based Complement Alternat Med* 2020**,** 8877998. doi: 10.1155/2020/8877998
